# N^7^-methylguanosine tRNA modification promotes esophageal squamous cell carcinoma tumorigenesis via the RPTOR/ULK1/autophagy axis

**DOI:** 10.1038/s41467-022-29125-7

**Published:** 2022-03-18

**Authors:** Hui Han, Chunlong Yang, Jieyi Ma, Shuishen Zhang, Siyi Zheng, Rongsong Ling, Kaiyu Sun, Siyao Guo, Boxuan Huang, Yu Liang, Lu Wang, Shuang Chen, Zhaoyu Wang, Wei Wei, Ying Huang, Hao Peng, Yi-Zhou Jiang, Junho Choe, Shuibin Lin

**Affiliations:** 1 Department of Otolaryngology, Center for Translational Medicine, Precision Medicine Institute, The First Affiliated Hospital, Sun Yat-sen University, Guangzhou, 510080 China; 2 Department of Thoracic Surgery, The First Affiliated Hospital, Sun Yat-sen University, Guangzhou, 510080 China; 3 Institute for Advanced Study, Shenzhen University, Shenzhen, 518057 China; 4 Department of Gastrointestinal Surgery, The First Affiliated Hospital of Sun Yat-sen University, 510080 Guangzhou, China; 5 Department of Pediatrics, The First Affiliated Hospital, Sun Yat-sen University, Guangzhou, 510080 China; 6 Department of Life Science, College of Natural Sciences, Hanyang University, Seoul, 04763 Republic of Korea; 7 Research Institute for Natural Sciences, Hanyang University, Seoul, 04763 Republic of Korea; 8 State Key Laboratory of Oncology in South China, Sun Yat-sen University Cancer Center, Guangzhou, 510060 China

**Keywords:** Post-translational modifications, Cancer

## Abstract

Mis-regulated RNA modifications promote the processing and translation of oncogenic mRNAs to facilitate cancer progression, while the molecular mechanisms remain unclear. Here we reveal that tRNA m^7^G methyltransferase complex proteins METTL1 and WDR4 are significantly up-regulated in esophageal squamous cell carcinoma (ESCC) tissues and associated with poor ESCC prognosis. In addition, METTL1 and WDR4 promote ESCC progression via the tRNA m^7^G methyltransferase activity in vitro and in vivo. Mechanistically, METTL1 or WDR4 knockdown leads to decreased expression of m^7^G-modified tRNAs and reduces the translation of a subset of oncogenic transcripts enriched in RPTOR/ULK1/autophagy pathway. Furthermore, ESCC models using *Mettl1* conditional knockout and knockin mice uncover the essential function of METTL1 in promoting ESCC tumorigenesis in vivo. Our study demonstrates the important oncogenic function of mis-regulated tRNA m^7^G modification in ESCC, and suggest that targeting METTL1 and its downstream signaling axis could be a promising therapeutic target for ESCC treatment.

## Abstract (web summary)

Deregulation of METTL1-mediated N7- methylguanosine tRNA modification can promote oncogenesis. Here, the authors report that this modification regulates the translation of proteins in both the mTOR and negative regulators of autophagy pathways, resulting in the progression of esophageal squamous cell carcinoma.

## Introduction

Esophageal cancer is an aggressive malignancy that causes over 400,000 deaths worldwide annually^1,2^. The two main subtypes of esophageal cancer, esophageal squamous cell carcinoma (ESCC) and adenocarcinoma (EAC), have different epidemiological distributions and pathogenesis mechanisms^3^. ESCC accounts for more than 70% of esophageal cancers and is related to extensive lymphatic spread and vascular invasion^4^. ESCC is usually diagnosed at advanced stages^5^. Though current therapeutic strategies including chemotherapy or surgical treatment could improve ESCC prognosis, the 5-year survival rate of ESCC patients still remains very low^6,7^. Thus, it is of great significance to identify effective therapeutic targets for better treatment of ESCC.

Transfer RNAs (tRNAs) are the modulators that recognize codons on mRNAs and bridge amino acids for mRNA translation^8^. tRNAs are subjected to extensive posttranscriptional modifications that are essential for tRNA folding, stability, and function^9,10^. Dysregulated tRNA modifications are frequently associated with developmental diseases and cancers^8,11^. N^7^-methylguanosine (m^7^G) is a common tRNA modification that is highly conserved in prokaryotes, eukaryotes, and some archaea^12^. Different from many essential tRNA modifications, the m^7^G tRNA modification is non-essential for yeast growth and survival under normal conditions, but is required for heat response in the yeast^13,14^. In human, tRNA m^7^G modification is catalyzed by the METTL1/WDR4 complex, and its mutations are associated with developmental disorders^15–17^, suggesting that tRNA m^7^G modification could have more important physiological functions in mammals. Our previous study revealed that METTL1 knockout decreases m^7^G tRNA modification and leads to impaired embryonic stem cell self-renewal and differentiation^18^, supporting the critical role of m^7^G tRNA modification in stem cells. In addition, recent studies also uncovered that METTL1 is upregulated in cancers and associated with chemosensitivity^19–28^, however, the detailed functions and molecular mechanisms of tRNA m^7^G modification in ESCC are still largely unknown.

In this study, we demonstrated that the METTL1 and WDR4 levels are significantly upregulated in ESCC and associated with poor ESCC prognosis. Dysregulated tRNA m^7^G modification upon depletion of METTL1/WDR4 inhibits ESCC initiation and progression in vitro and in mouse models. Mechanically, tRNA m^7^G modification regulates the translation of m^7^G-related codon enriched mRNAs including mTOR and negative regulators of autophagy pathway genes. Our work provides a link between tRNA modification, mTOR pathway, autophagy, and ESCC progression, which might shed light on the development of promising targeted drugs for ESCC therapy.

## Results

### METTL1/WDR4 are upregulated in ESCCs and associated with poor ESCC prognosis

To explore the role of m^7^G tRNA modification in cancer, we first analyzed The Cancer Genome Atlas (TCGA) datasets and revealed that both METTL1 and WDR4 mRNAs are elevated in multiple cancers (Supplementary Fig. 1a, b). Especially, METTL1 and WDR4 showed dramatic upregulation in Esophageal Carcinoma (TCGA-ESCA) (Supplementary Fig. 1a, b). Further analysis revealed that METTL1 and WDR4 are upregulated in esophageal adenocarcinoma (EAC) samples and further increased in esophageal squamous cell carcinoma (ESCC) samples (Supplementary Fig. 1c, d). To explore the potential mechanisms underlying METTL1/WDR4 overexpression in ESCCs, we first analyzed the METTL1 and WDR4 genome and found that small percentage of ESCC patients contain DNA amplification, mutation, or deletion on METTL1 or WDR4 genes (Supplementary Fig. 1e). Moreover, the expression levels of METTL1 and WDR4 are negatively associated with their DNA methylation statuses and patient age (Supplementary Fig. 1f–i). Correlation analysis showed that the expression levels of METTL1 and WDR4 are positively correlated in ESCCs (Supplementary Fig. 1j), suggesting the potential roles of METTL1 and WDR4 in regulation of ESCC progression. Overall, these data revealed that both genetic and epigenetic mechanisms could lead to the aberrant METTL1 and WDR4 expression in ESCC patients.

**Fig. 1:**
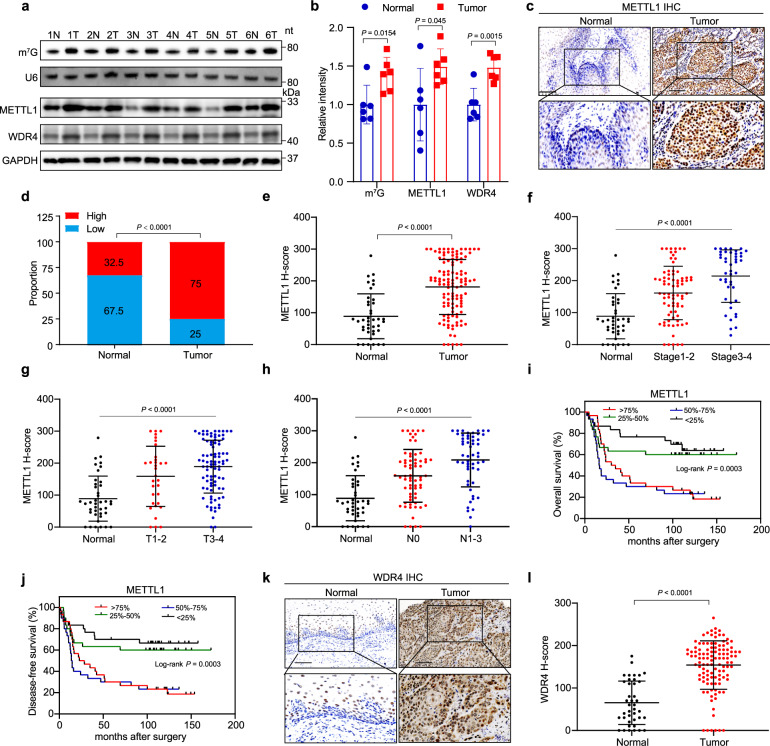
METTL1 is elevated in ESCCs and is a negative prognostic factor for ESCC patients. **a**, **b** Blotting assays (**a**) and the quantitative analysis (**b**) of METTL1, WDR4, and m^7^G levels in ESCC tumors and adjacent control normal tissues. N, Normal. T, Tumor. **c** Representative images of METTL1 IHC staining in esophageal tumor tissues and adjacent normal tissues. Scale bar, 100 μm. **d** Proportion of METTL1 expression cases in adjacent normal tissues and esophageal tumor tissues. **e** Quantification of METTL1 H-scores in esophageal tumor tissues and adjacent normal tissues. **f** Quantification of METTL1 H-scores in adjacent normal tissues and different stages of esophageal tumor tissues. **g** Quantification of METTL1 H-scores in adjacent normal tissues and different grades of esophageal tumor tissues. **h** Quantification of METTL1 H-scores in adjacent normal tissues and esophageal tumor tissues with different stages of lymph node metastasis. **i**, **j** Kaplan–Meier analysis of the overall survival (**i**) and disease-free survival (**j**) of ESCC patients based on H-scores of METTL1. **k** Representative images of WDR4 IHC staining in esophageal tumor tissues and adjacent normal tissues. **l** Quantification of WDR4 H-scores in esophageal tumor tissues and adjacent normal tissues. Data are presented as mean ± SD from six independent patient samples for (**b**) and 120 patient samples for (**c**, **e**–**h**, and **l**). *P* values are indicated by two-tailed unpaired Student’s *t* test for (**b**), Pearson chi-square test for (**d**), Mann–Whitney U test for (**e**, **l**), Kruskal–Wallis test for (**f**–**h**), and log-rank test for (**i**, **j**). Source data are provided as a Source data file. The source data of (**i**, **j**) are protected and are not available due to data privacy laws.

To verify the above results, we examined METTL1/WDR4 expression using our cohort of ESCC samples. Our data showed that METTL1/WDR4 expression and m^7^G tRNA modification were elevated in ESCC samples compared to their corresponding controls (Fig. 1a, b). In addition, Immunohistochemistry (IHC) staining revealed the increased METTL1 expression in ESCC tumors than that in peri-tumor tissues (Fig. 1c–e). Moreover, the expression of METTL1 is significantly associated with high tumor grades and stages (Fig. 1f–h), suggesting the essential function of METTL1 in ESCC progression. We further examined the relationship between METTL1 expression and the prognosis of ESCC patients. Survival analysis showed that high METTL1 expression is correlated with poor overall survival and disease-free survival status (Fig. 1i, j). Similarly, WDR4 is also upregulated in ESCCs and associated with poor prognosis of ESCC patients (Fig. 1k, l, Supplementary Fig. 1k, l). Overall, our data revealed that METTL1 and WDR4 are significantly upregulated in ESCCs and associated with ESCC progression and poor prognosis.

### Targeting METTL1 expression inhibits ESCC progression

**Fig. 2:**
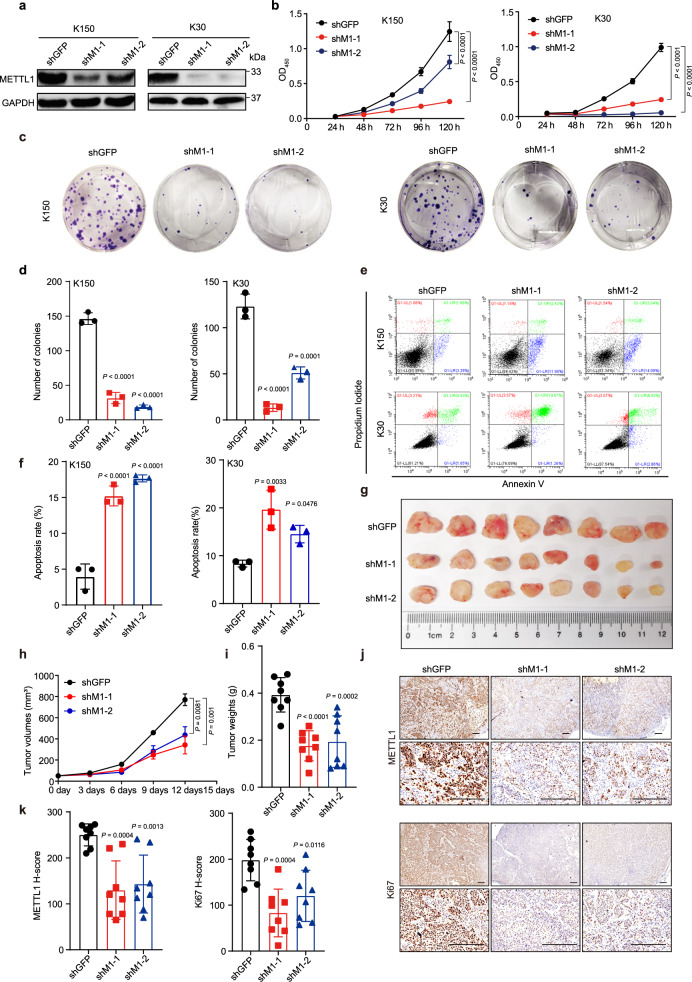
METTL1 knockdown impairs ESCC progression in vitro and in xenograft model. **a** Western blotting of METTL1 in K150 and K30 ESCC cells. **b** Cell Counting Kit-8 assay (CCK8) of cell growth with METTL1 knockdown and control cells. **c**, **d** Colony-formation assay (**c**) and the quantitative analysis (**d**) of METTL1 knockdown and control cells. **e**, **f** Apoptosis assay (**e**) and the quantitative analysis (**f**) of METTL1 knockdown and control cells. **g** Overview of tumors in xenograft mice model subcutaneously implanted with METTL1 knockdown and control K150 ESCC cells. **h** Growth curves of tumor volumes in METTL1 knockdown and control K150 ESCC cells. **i** Tumor weights in METTL1 knockdown and control K150 ESCC cells. **j**, **k** Representative images of METTL1 and Ki67 IHC staining (**j**) and the quantitative H-scores (**k**) of tumors obtained from the K150 xenograft model. Scale bar, 200 μm. Data represented as mean ± SD from three independent experiments for (**b**, **d**, and **f**), and eight biological independent samples for (**h**–**k**). Significant difference from control group calculated by one-way ANOVA with Dunnett’s multiple comparison test was indicated on graph. Source data are provided as a Source data file.

The elevated METTL1 expression in the ESCC led us to hypothesize that METTL1 plays an important oncogenic function in ESCC progression. To test this hypothesis, we first knocked down METTL1 expression using two short hairpin RNAs (shM1-1 and shM1-2) in KYSE150 (K150) and KYSE30 (K30) ESCC cells (Fig. 2a). Knockdown of METTL1 inhibited the proliferation (Fig. 2b, Supplementary Fig. 2a, b) and colony-formation ability (Fig. 2c, d) of both K150 cells and K30 cells. Moreover, flow cytometry analysis revealed that shM1-1 and shM1-2 resulted in increased apoptosis of ESCC cells (Fig. 2e, f, Supplementary Fig. 2c). Then, we used xenograft mouse model to explore the function of METTL1 in ESCC progression in vivo. Mice injected with METTL1 knockdown cells showed significantly slower tumor growth, reduced tumor sizes and weights compared with those injected with shGFP ESCC cells (Fig. 2g–i, Supplementary Fig. 2d–f). IHC staining showed the decreased METTL1 expression and reduced Ki67 staining level in tumors of the shM1-1 and shM1-2 groups, confirming that METTL1 knockdown reduced the proliferative activity of ESCC in vivo (Fig. 2j, k, Supplementary Fig. 2g, h). Taken together, our studies revealed the essential functions of METTL1 in ESCC progression in vitro and in vivo.

### Inhibition of WDR4 suppresses ESCC progression

To further verify the role of tRNA m^7^G modification in ESCC progression, we generated the WDR4 knockdown (shW4-1 and shW4-2) K150 and K30 cells (Supplementary Fig. 3a) and examined the effects of WDR4 knockdown on ESCC progression. Similar to METTL1 knockdown, loss of WDR4 led to slower cell growth, less colony formation, and increased apoptosis in both K150 and K30 ESCC cells (Supplementary Fig. 3b–f). We further examined the effect of WDR4 knockdown on ESCC progression in vivo using the subcutaneous xenograft model. Depletion of WDR4 led to significantly slower tumor growth, reduced tumor sizes and weights in vivo (Supplementary Fig. 3g–i). IHC staining confirmed the decreased WDR4 expression and reduced proliferative activity in the tumors from the shW4-1 and shW4-2 groups (Supplementary Fig. 3j, k). Our data further supported the essential function of m^7^G tRNA modification in ESCC progression.

### METTL1 regulates m^7^G tRNA modification, tRNA expression, and mRNA translation in ESCCs

**Fig. 3:**
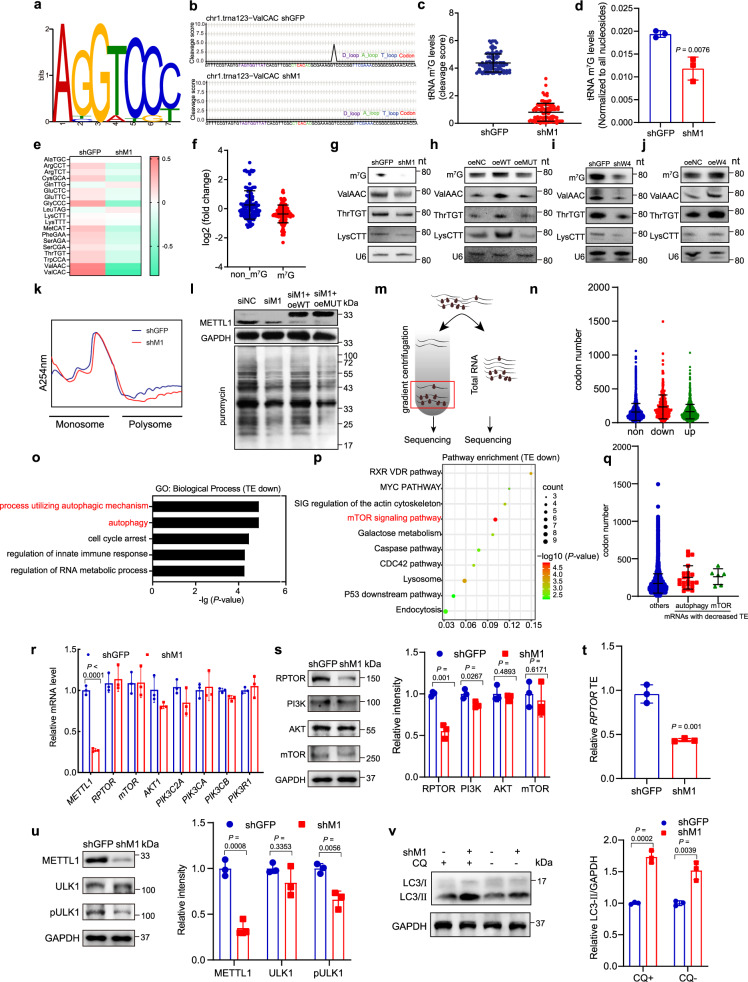
METTL1 regulates tRNA m^7^G modification, tRNA expression, and oncogenic mRNA translation. **a** Motif sequence at m^7^G site. **b** Representative image of cleavage score. **c** Quantification of m^7^G level on m^7^G-modified tRNAs. **d** LC-MS-based detection of m^7^G tRNA modification levels on tRNAs. **e** Expression profile of m^7^G-modified tRNAs in the METTL1 knockdown and control cells. Each cell shows the relative expression of all isodecoders of a specific tRNA type. Expression of each tRNA type was normalized by its overall average level in both groups and transformed by log2. **f** Mann–Whitney U test on the expression of the m^7^G-modified and non-m^7^G tRNAs. **g**–**j** Northern blot of indicated tRNAs. U6 snRNA was used as a loading control. **k** Polysome profiling of METTL1 depleted and control K150 cells. **l** Puromycin intake assay of K150 cells. **m** Schematic of polyribosome-seq. **n** The numbers of m^7^G tRNA-decoded codons in mRNAs with upregulated TEs (up), downregulated TEs (down), and unaltered TEs (non) in the METTL1 depleted cells. **o** Gene ontology analysis using the TE-down genes. **p** Pathway analysis using the TE-down genes. **q** m^7^G tRNA-decoded codon numbers in TE-decreased mRNAs enriched in the negative regulation of autophagy or mTOR signaling pathway and other mRNAs. **r** qRT-PCR analysis of representative genes in K150 cells. **s** Western blot analysis of indicated proteins in K150 cells, right panel: quantification of the western blot signals. **t** qRT-PCR based TE analysis of *RPTOR* using the polyribosome mRNAs. **u** Western blot analysis of ULK1 and pULK1 in K150 cells, right panel: quantification of the western blot signals. **v** Western blot analysis of LC3 in K150 cells, right panel: quantification of the western blot signals. Data presented as mean ± SD from two independent biological samples for each group for (**c**, **e**, and **f**), three independent biological samples for (**d**), one biological sample for (**n**, **q**), and three independent experiments for (**r**–**v**). *P* values are calculated by Mann–Whitney U test for (**c**, **f**, **n**, and **q**), and two-tailed unpaired Student’s *t* test for (**d**, **r**, **s**, **t**, **u**, and **v**). Source data are provided as a Source data file.

To study the molecular mechanisms underlying METTL1’s function in ESCC progression, we profiled the global tRNA m^7^G modifications using the METTL1 depleted and control ESCC cells using our previously established TRAC-seq (tRNA reduction and cleavage sequencing) method^18,29^. Nineteen m^7^G-modified tRNAs with “ABGWY” motif sequences in the variable loop were identified in our TRAC-seq data (Fig. 3a). METTL1 depletion significantly reduced m^7^G modification level, as demonstrated by the reduced cleavage scores (Fig. 3b, c). Our RNA mass spectrometry also confirmed the decreased tRNA m^7^G modification levels in the METTL1 knockdown cells (Fig. 3d). Moreover, depletion of METTL1 reduced the expression levels of the majority of m^7^G-modified tRNAs, while the expression of the non-m^7^G-modified tRNAs was barely affected (Fig. 3e, f). Consistent with the TRAC-seq data, our m^7^G northwestern and northern blot assays confirmed the reduced m^7^G modification levels and decreased expression of the m^7^G-modified tRNAs in METTL1 knockdown cells (Fig. 3g and Supplementary Fig. 4a). In addition, overexpression of wild-type METTL1 but not its catalytic dead mutant increased the global m^7^G tRNA modification levels and the expression of m^7^G modified tRNAs (Fig. 3h and Supplementary Fig. 4b), confirming that METTL1 regulates tRNA modification and expression through its tRNA m^7^G methyltransferase activity. Similarly, inhibition of WDR4 led to decreased m^7^G modification level and m^7^G-modified tRNA expression; and overexpression of WDR4 increased m^7^G tRNA modification and m^7^G-modified tRNA expression in both K150 and K30 ESCC cells (Fig. 3i, j and Supplementary Fig. 4c, d). These data supported the essential function of METTL1/WDR4 in the regulation of tRNA m^7^G modification and expression.

Given that tRNAs function in mRNA translation, we next determined the effect of METTL1 on mRNA translation in ESCC cells. Polysome profiling assay showed that the mRNA translation activity was reduced in the METTL1 knockdown cells, as reflected by the decreased polyribosome peak (Fig. 3k and Supplementary Fig. 4e). In addition, puromycin intake assay also confirmed that METTL1 knockdown decreased mRNA translation activity in ESCC cells (Fig. 3l and Supplementary Fig. 4f). Re-expression of wild-type METTL1 but not its mutant rescued the mRNA translation efficiency in the METTL1 depleted cells (Fig. 3l and Supplementary Fig. 4f), proving that the m^7^G catalytic function is essential for METTL1 to promote mRNA translation. Collectively, our data demonstrated that METTL1-mediated m^7^G tRNA modification is critical for tRNA expression and mRNA translation in ESCC.

### METTL1 regulates the translation of mRNAs with high number of m^7^G-related codons

To study the effect of translation disorder mediated by abnormal m^7^G modification, we performed polyribosome-bound mRNAs sequencing (polyribosome-seq) (Fig. 3m) using the METTL1 knockdown and control ESCC cells. To investigate the link between the mRNA translation and tRNA m^7^G modification, we calculated the number of the codons decoded by the m^7^G-modified tRNAs on differentially translated mRNAs. Our data revealed that the mRNAs with reduced translation efficiencies (TEs) have notably larger number of codons decoded by m^7^G-modified tRNAs (Fig. 3n). Gene ontology analysis and pathway analysis showed that TE-down mRNAs are significantly enriched in autophagic biological process and mTOR signaling pathway (Fig. 3o, p and Supplementary Table 1). Notably, those mRNAs with decreased TEs and involved in autophagic biological process or mTOR signaling pathway have larger number of codons decoded by m^7^G-modified tRNAs than other detected mRNAs (Fig. 3q). Furthermore, our ribosome profiling sequencing (Ribo-seq) data showed that mRNAs with decreased translation efficiencies have larger number of m^7^G-tRNA-dependent codons (Supplementary Fig. 4g, h), and METTL1 knockdown promoted ribosome pausing at codons decoded by m^7^G tRNAs (Supplementary Fig. 4i), suggesting that METTL1-mediated m^7^G tRNA modification is essential for ribosome transition on m^7^G codons. Gene set enrichment analysis (GSEA) of the differentially translated mRNAs identified by Ribo-seq also revealed that METTL1 regulates the translation of transcripts related to negative regulation of autophagy and mTOR signaling pathway (Supplementary Fig. 4j).

We next determined METTL1’s function in regulating the expression of negative regulation of autophagy and mTOR signaling pathway genes. Our data revealed that METTL1 depletion reduced their protein expression but had little impact on their mRNAs (Fig. 3r, s). In addition, polyribosome-qPCR assay further confirmed that the translation efficiency of *RPTOR* (Regulatory Associated Protein of mTOR Complex 1), which encodes a key mTOR component with a high abundance of m^7^G tRNA-decoded codons (Supplementary Fig. 4k), was remarkably decreased in METTL1 depleted cells (Fig. 3t and Supplementary Fig. 4l). The mTOR signaling pathway negatively regulates autophagy by phosphorylating ser758 of ULK1 (pULK1)^30^. Therefore, we then determined the levels of pULK1 and the autophagy marker microtubule-associated protein light chain 3, LC3-II and LC3-I, in the METTL1 knockdown cells. METTL1 depletion reduced the level of pULK1, and increased the ratio of LC3-II/LC3-I in both chloroquine (CQ) treated and untreated K150 and K30 cells (Fig. 3u, v and Supplementary Fig. 4m), suggesting the increased autophagy level in the METTL1 knockdown ESCC cells. Interestingly, METTL1 depletion resulted in the impairment of overall mRNA translation level and reduced RPTOR expression within 24 h (Supplementary Fig. 5a, b), while increased cell death and autophagy occurred at 48 h after METTL1 depletion (Supplementary Fig. 5c, d). These data indicated that the translation impairment occurs prior to cell death and autophagy, suggesting that METTL1 knockdown decreases mRNA translation, which then leads to cell death and autophagy in ESCC cells. Taken together, our data demonstrated that METTL1 promotes the translation of genes related to mTOR signaling and negative regulation of autophagy pathway in an m^7^G-related codon-dependent manner.

### m^7^G-modified tRNAs mediate METTL1’s function in ESCC progression

Given that METTL1 knockdown decreases the expression of m^7^G-modified tRNAs and inhibits ESCC progression, we further investigated whether m^7^G-modified tRNAs facilitate METTL1’s function in promoting ESCC progression. We overexpressed ValAAC or/and LysCTT tRNAs to perform rescue assay because they are downregulated by METTL1 depletion (Fig. 3g), and their decoded codons are highly abundant in the TE-down mRNAs (Supplementary Fig. 6a). Our data revealed that the ectopically overexpressed tRNAs were sufficiently charged and could partially rescue RPTOR protein expression in the METTL1 depleted cells (Supplementary Fig. 6b–d). Functionally, overexpression of ValAAC or LysCTT separately could partially rescue the cell growth in the METTL1 depleted cells, while overexpression of both ValAAC and LysCTT simultaneously showed a synergistic effect in rescuing the cell proliferation in METTL1 depleted cells (Supplementary Fig. 6e). These data further support that m^7^G modified tRNAs are essential downstream targets that facilitate METTL1’s function in ESCC progression.

### RPTOR is an essential downstream target of METTL1 in ESCC

**Fig. 4:**
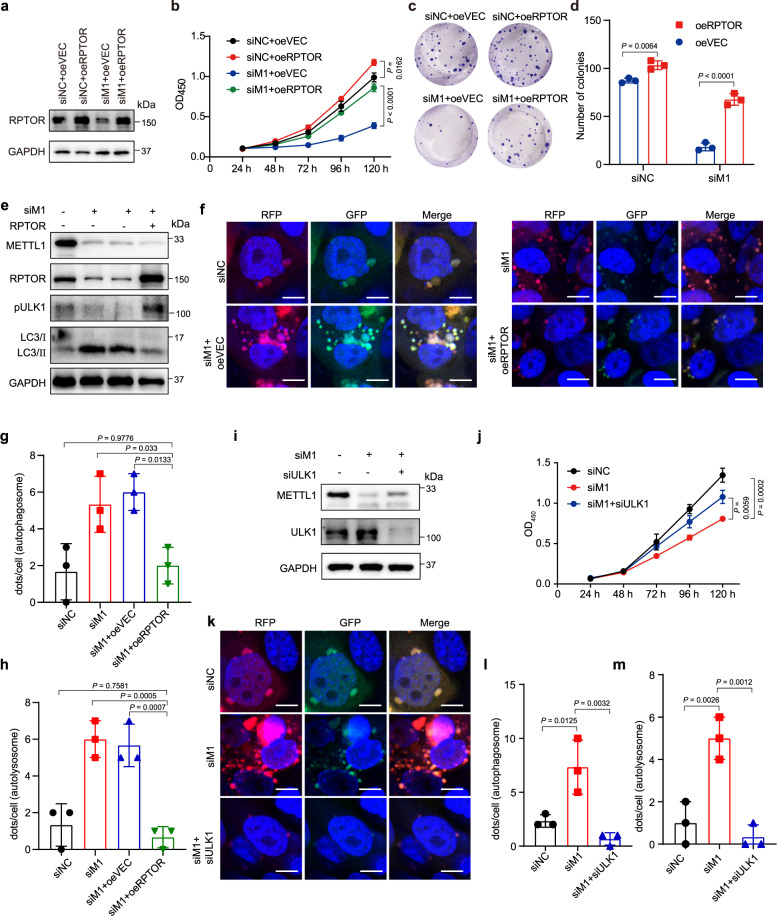
METTL1/RPTOR/ULK1 axis plays essential roles in ESCC progression. **a** Western blot analysis with indicated antibodies in METTL1 knockdown K150 cells with or without RPTOR overexpression. **b** CCK8 assay of METTL1 knockdown K150 cells with or without RPTOR overexpression. **c**, **d** Colony-formation assay (**c**) and the quantification analysis (**d**) of METTL1 knockdown K150 cells with or without RPTOR overexpression. **e** Western blot analysis with indicated antibodies in METTL1 knockdown K150 cells with or without RPTOR overexpression. **f**–**h** The autophagic fluxes (**f**) and the quantification analysis (**g**, **h**) of K150 cells stably expressed mRFP-EGFP-LC3 fusion protein. Scale bar, 10 μm. **i** Western blot analysis with indicated antibodies. **j** CCK8 assay of METTL1 knockdown K150 cells with or without ULK1 knockdown. **k**–**m** The autophagic fluxes (**k**) and the quantification analysis (**l**, **m**) of K150 cells stably expressed mRFP-EGFP-LC3 fusion protein. Scale bar, 10 μm. Data represented as mean ± SD from three independent experiments. *P* values are presented by one-way ANOVA with Tukey’s multiple comparison test for (**b**, **d**), and one-way ANOVA with Dunnett’s multiple comparison test for (**g**, **j**–**h**, and **l**, **m**).

To verify the role of RPTOR/ULK1/autophagy axis in METTL1’s function in ESCC, we performed rescue assay to overexpress RPTOR in METTL1 knockdown ESCC cells. We found that overexpression of RPTOR could increase p4EBP1 and pS6K1 levels and rescue the mRNA translation activity in METTL1 depleted cells (Supplementary Fig. 7a, b). Translation profiling revealed that RPTOR overexpression increased the translation of a subset of mRNAs in METTL1 depleted cells (Supplementary Fig. 7c). We then compared the METTL1-dependent and RPTOR-dependent mRNAs and found m^7^G codon enrichment in METTL1-dependent mRNAs but not in the RPTOR-dependent mRNAs (Supplementary Fig. 7d, e). We further revealed that re-expression of RPTOR rescued the proliferation and colony-formation capacities in METTL1 depleted ESCC cells (Fig. 4a–d, Supplementary Fig. 8a–d). Moreover, RPTOR overexpression increased the phosphorylation level of ULK1, reduced the ratio of LC3-II/LC3-I, and eliminated autophagic flux in METTL1 depleted cells ESCC cells (Fig. 4e–h, Supplementary Fig. 8a, e–g). Overall, these data revealed that RPTOR is an essential downstream target of METTL1 and further supported that METTL1-mediated tRNA m^7^G modification promotes ESCC progression through regulation of RPTOR.

### Negative regulators of autophagy mediate METTL1 and RPTOR’s function in ESCC progression

Given that RPTOR could rescue the phosphorylation level of ULK1 and reduce autophagy in METTL1 knockdown ESCC cells, we next knocked down ULK1 in METTL1 depleted ESCC cells to determine whether METTL1 and RPTOR promote ESCC progression via regulation of ULK1-mediated autophagy (Fig. 4i and Supplementary Fig. 8h). Our data demonstrated that inhibition of ULK1 could rescue the growth in METTL1 depleted ESCC cells (Fig. 4j and Supplementary Fig. 8i), further demonstrating that the METTL1 and RPTOR regulate ESCC progression through ULK1. Moreover, autophagic flux detection showed that knockdown of ULK1 could eliminate the increased autophagic flux in METTL1 depleted ESCC cells (Fig. 4k–m and Supplementary Fig. 8j–l). Given that ULK1 has autophagy-independent functions^31^, we further manipulated two other autophagy genes (*VPS34* and *BECN1*) in the METTL1 knockdown ESCC cells and revealed that knockdown of VPS34 or BECN1 could partially rescue the ESCC progression in METTL1 depleted cells (Supplementary Fig. 9a–d). Overall, our data uncovered the essential roles of RPTOR/autophagy axis in mediating METTL1’s function in ESCC progression.

### Conditional knockout (cKO) of *Mettl1* inhibits ESCC tumorigenesis in vivo

**Fig. 5:**
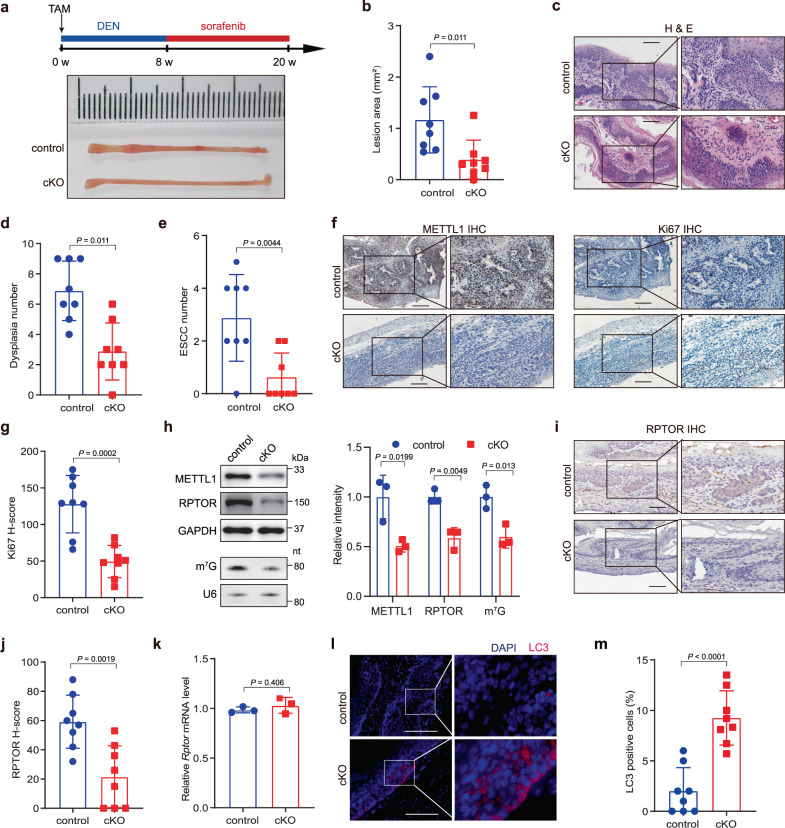
Knockout of *Mettl1* inhibits in vivo ESCC tumorigenesis. **a** Experimental design for ESCC tumorigenesis model and the representative images of esophageal lesions from *Mettl1* cKO and control mice. **b** Quantification of lesion areas in *Mettl1* cKO and control mice. **c** Representative H&E staining of esophagus tissues in *Mettl1* cKO and control mice. Scale bar, 100 μm. **d** Quantification of dysplasia numbers in *Mettl1* cKO and control mice. **e** Quantification of ESCC numbers in *Mettl1* cKO and control mice. **f**, **g** Representative images of METTL1 and Ki67 IHC staining (**f**) and the quantification of Ki67 H-scores (**g**) in *Mettl1* cKO and control mice. Scale bar, 100 μm. **h** Western blot analysis and northwestern blot analysis showed the m^7^G modification level and indicated protein levels, right panel: quantification of the blot signals, *n* = 3 biological independent samples for each group. **i**, **j** Representative images (**i**) and the quantification of H-scores (**j**) of RPTOR IHC staining in *Mettl1* cKO and control mice. Scale bar, 100 μm. **k** qRT-PCR analysis of *Rptor* mRNA level in cKO and control mice, *n* = 3 biological independent samples for each group. **l**, **m** IF assay (**l**) and the quantification (**m**) of LC3 levels in *Mettl1* cKO and control mice. Scale bar, 100 μm. Data represented as mean ± SD by two-tailed unpaired Student’s *t* test for all. *n* = 8 mice for each group for (**a**–**g**, **i**, **j**, and **l**, **m**).

To directly explore the function of m^7^G tRNA modification in ESCC tumorigenesis in vivo, we generated the epithelial tissue-specific *Mettl1* conditional knockout (*Keratin14-CreER; Mettl1*^*fl/fl*^, cKO) mouse model. After induction of *Mettl1* knockout using tamoxifen, the cKO and control mice were treated with DNA alkylating agent diethylnitrosamine (DEN) and multikinase inhibitor sorafenib to induce ESCC tumorigenesis^32^ (Fig. 5a). After DEN treatment for 8 weeks followed by sorafenib treatment for 12 weeks, we found remarkable pathological changes in the esophagus of control mice, while the lesion area and ESCC numbers were significantly reduced in the *Mettl1* cKO mice (Fig. 5a–e). Histological analysis revealed that the tumors in cKO mice showed decreased levels of METTL1 and Ki67 staining compared with the tumors in control mice (Fig. 5f, g). We also found that *Mettl1* cKO reduced m^7^G tRNA modification and decreased RPTOR protein level in the tumors of *Mettl1* cKO mice (Fig. 5h–j), while the mRNA level of RPTOR remained unchanged (Fig. 5k). In addition, the levels of pULK1, pS6K1, and p4EBP1 were reduced in the tumors of *Mettl1* cKO mice (Supplementary Fig. 10a–f). These data supported that METTL1 and m^7^G tRNA modification regulated RPTOR mRNA translation and the activity of RPTOR downstream targets in vivo. Notably, the protein level of LC3 was significantly higher in tumors in cKO group than those in control group (Fig. 5l, m), suggesting that *Mettl1* knockout leads to increased autophagy in vivo. Overall, these results support the critical function of METTL1-mediated m^7^G tRNA modification in promoting in vivo ESCC tumorigenesis.

### Knockout of *Mettl1* suppresses ESCC progression in vivo

To study the effect of METTL1 on ESCC progression in vivo, we first induced ESCC formation in the *Mettl1* cKO and control mice, then the tumor-bearing mice were treated with tamoxifen to induce *Mettl1* knockout in the cKO mice (Supplementary Fig. 11a). Our data showed that knockout of *Mettl1* in the tumor-bearing mice significantly decreased the lesion area and numbers of dysplasia and ESCC (Supplementary Fig. 11b–e). Histological analysis showed the significantly decreased levels of METTL1 and Ki67 staining in cKO mice, confirming that knockout of *Mettl1* reduces ESCC proliferation in vivo (Supplementary Fig. 11f, g). In addition, knockout of *Mettl1* in the pre-existing ESCCs decreased m^7^G tRNA modification, RPTOR protein level and increased the level of LC3 in the ESCC tumors (Supplementary Fig. 11h–m), supporting the essential roles of METTL1/m^7^G tRNA modification/RPTOR/autophagy axis in ESCC progression in vivo.

### METTL1/WDR4 mediated m^7^G tRNA modification promotes ESCC progression

**Fig. 6:**
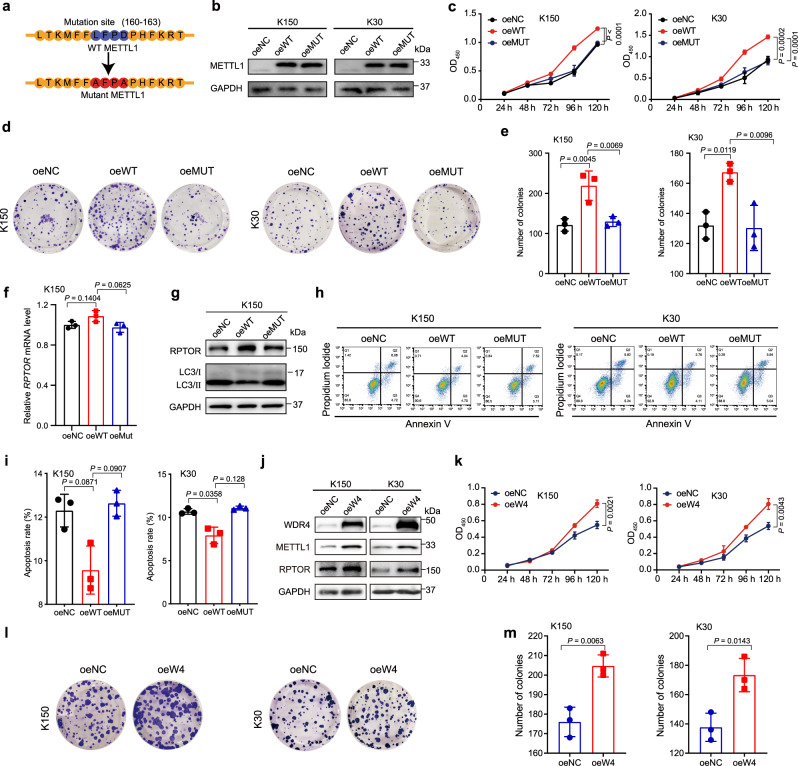
Overexpression of METTL1 or WDR4 promotes ESCC progression. **a** The catalytic mutation site of METTL1. **b** Western blot confirmed the overexpression of wild-type METTL1 (oeWT) and catalytic inactive METTL1 (oeMUT) compared with the negative control (oeNC) in ESCC cells. **c** CCK8 assay of growth of oeNC, oeWT, and oeMUT ESCC cells. **d**, **e** Colony-formation assay (**d**) and the quantification analysis (**e**) of oeNC, oeWT, and oeMUT ESCC cells. **f** qRT-PCR assay of RPTOR mRNA level. **g** Western blot assay showed the indicated protein levels. **h**, **i** Apoptosis assay (**h**) and the quantitative analysis (**i**) of METTL1 overexpression and control cells. **j** Western blot assay showed the indicated protein levels. **k** CCK8 assay of growth of oeNC and oeWDR4 ESCC cells. **l**, **m** Colony-formation assay (**l**) and the quantification analysis (**m**) of oeNC and oeWDR4 ESCC cells. Data represented as mean ± SD from three independent experiments. *P* values are presented by one-way ANOVA with Dunnett’s multiple comparison test for (**c**, **e**, **f**, and **i**), and two-tailed unpaired Student’s *t* test for (**k** and **m**).

To further strengthen the functional link between m^7^G tRNA modification and ESCC progression, we performed gain-of-function studies (Fig. 6a). Our data revealed that forced expression of wild-type METTL1 but not its catalytic dead mutant could promote ESCC cell growth and colony-formation (Fig. 6b–e). Furthermore, overexpression of METTL1 but not its mutant upregulated RPTOR protein expression and reduced cellular apoptosis and autophagy (Fig. 6f–i). Overall, our data revealed that the tRNA m^7^G catalytic function is essential for METTL1 in regulating target expression and ESCC progression.

We then overexpressed WDR4 in ESCC cells to further confirm our conclusion that m^7^G tRNA modification promotes ESCC progression. Our data revealed that overexpression of WDR4 stabilized METTL1 protein expression and increased the expression of downstream target RPTOR (Fig. 6j). Functional analysis revealed that forced expressed WDR4 could promote ESCC cell growth and colony-formation (Fig. 6k–m). These data supported that WDR4 is an important oncogene promoting the ESCC progression. Overall, our results strongly supported the essential oncogenic function of METTL1/WDR4 mediated m^7^G tRNA modification in regulation of ESCC progression.

### METTL1 promotes ESCC tumorigenesis in vivo

**Fig. 7:**
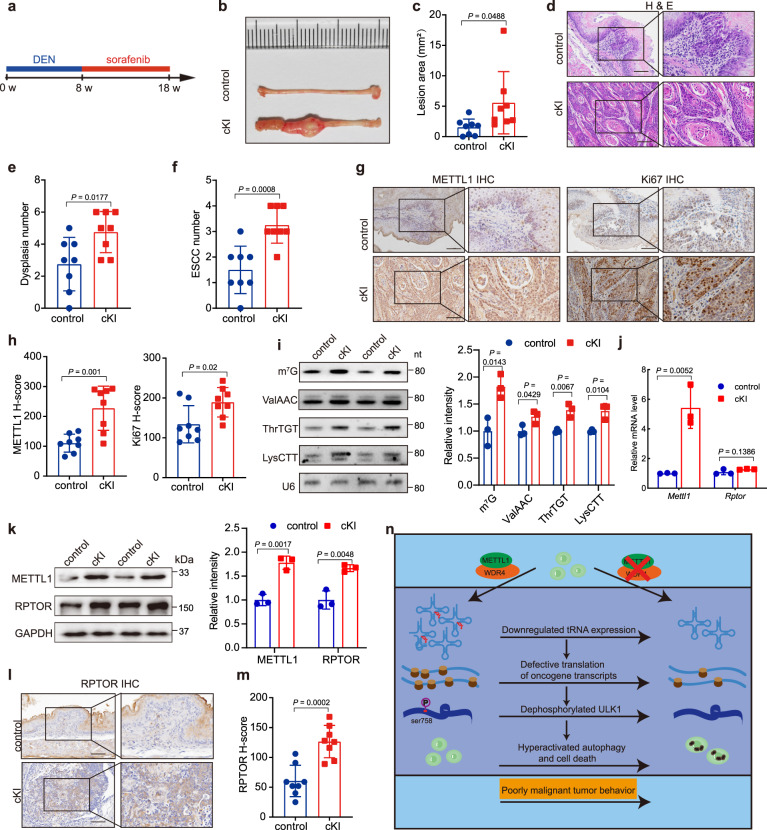
METTL1 overexpression promotes in vivo ESCC tumorigenesis. **a** Experimental design for ESCC tumorigenesis model in *Mettl1* cKI and control mice. **b** Representative images of esophageal lesions from *Mettl1* cKI and control mice. **c** Quantification of lesion areas in *Mettl1* cKI and control mice. **d** Representative H&E staining of esophagus tissues in *Mettl1* cKI and control mice. Scale bar, 100 μm. **e**, **f** Quantification of dysplasia numbers (**e**) and ESCC numbers (**f**) in *Mettl1* cKI and control mice. **g**, **h** Representative images (**g**) and the quantification (**h**) of METTL1 and Ki67 IHC staining in *Mettl1* cKI and control mice. Scale bar, 100 μm. **i** Northwestern blot and northern blot detected the m^7^G level and m^7^G-modified tRNA expression level in tumors from *Mettl1* cKI and control mice, right panel: quantification of the blot signals. **j** qRT-PCR assay showed the mRNA levels of indicated mRNAs. **k** Western blot showed the indicated protein levels in the cKI and control mice, right panel: quantification of the western blot signals. **l**, **m** Representative images (**l**) and the quantification (**m**) of RPTOR IHC staining in *Mettl1* cKI and control mice. Scale bar, 100 μm. **n** Working model for METTL1/WDR4 mediated tRNA m^7^G modification in regulation of ESCC tumorigenesis. Data represented as mean ± SD by two-tailed unpaired Student’s *t* test for all. *n* = 3 biological independent samples for (**i**–**k**) and *n* = 8 biological independent samples for (**b**–**h** and **l**, **m**).

We next determined the role of METTL1 overexpression in ESCC tumorigenesis in vivo. Xenograft mouse model revealed that METTL1 overexpression significantly promoted the ESCC progression in vivo, as revealed by the increased tumor volumes/weights and upregulated Ki67 levels (Supplementary Fig. 12a–f). We further established the *Mettl1* conditional knockin (*Keratin14-CreER; Mettl1*^*ki*^, cKI) mouse model and induced ESCC tumorigenesis to investigate the role of METTL1 in ESCC tumorigenesis in vivo. After DEN/sorafenib treatment for 18 weeks (Fig. 7a), massive tumor burden was developed in esophagus of the *Mettl1* cKI mice, while only few and small lesions occurred in the esophagus of control mice (Fig. 7b, c). In addition, the cKI mice had significantly increased number of dysplasia and squamous cell carcinoma than the control mice (Fig. 7d–f). Histological analysis revealed that the tumors in cKI mice showed higher METTL1 expression level, higher proliferative activity than those in control mice (Fig. 7g, h). We further revealed that m^7^G tRNA modification and the expression of m^7^G-modified tRNAs were elevated in tumors from cKI mice compared to those from control mice (Fig. 7i). Moreover, the RPTOR protein level but not its mRNA level increased in the tumors from *Mettl1* cKI mice (Fig. 7j–m). In addition, tumors from the *Mettl1* cKI mice also showed upregulated levels of pULK1, pS6K1, and p4EBP1 (Supplementary Fig. 10g–l), suggesting that METTL1 and m^7^G tRNA modification promoted RPTOR mRNA translation and the activity of its downstream targets in vivo. Taken together, these results revealed that METTL1 and m^7^G tRNA modification facilitates ESCC tumorigenesis and development in vivo.

## Discussion

Emerging evidence revealed that mRNA and protein levels are poorly correlated in cancers, and the cancer cells selectively promote the mRNA translation of oncogenes to enhance cancer transformation and progression^33,34^. Therefore, targeting the aberrant mRNA translation is a promising strategy for cancer therapy. tRNAs are essential adapters for mRNA translation and heavily modified, while the role and mechanisms of tRNA modifications in gene expression regulation and cancer progression are still poorly understood. In yeast, knockout of m^7^G tRNA methyltransferase Trm8 has no effect on yeast growth and survival under normal culture conditions, while the Trm8 knockout yeast cannot grow under heat stress, suggesting that the m^7^G tRNA modification is a non-essential modification that is only required for stress response in yeast^13,14^. Our previous study revealed that METTL1-mediated tRNA m^7^G modification is required for mouse embryonic stem cells self-renewal and differentiation^18^, suggesting that tRNA m^7^G modification could have more important physiological function in mammals than in the yeast. Interestingly, here we found that the expression of m^7^G tRNA methyltransferase METTL1 is low in normal tissues but significantly upregulated in the ESCC tumor samples, and the high expression of METTL1 is associated with poor patient prognosis. Functionally, knockdown of METTL1 or WDR4 strongly inhibits the proliferation of ESCC cells, and suppresses the in vivo ESCC growth in xenograft mouse model. Most importantly, our in vivo ESCC initiation and progression assays using the *Mettl1* conditional knockout and conditional knockin mice provided strong and direct evidence to support the important oncogenic role of METTL1 and tRNA m^7^G modifications in ESCC initiation and progression.

Mechanistically, depletion of METTL1 leads to decreased m^7^G modified tRNA expression, and reduced translation of mRNAs in an m^7^G-related codon-dependent manner. Gene set enrichment assay revealed that mRNAs with decreased translation efficiency are significantly enriched in mTOR signaling pathway and negative regulation of autophagy, suggesting that tRNA m^7^G modification regulates mTOR and autophagy in the ESCC cells. tRNAs are the dominant m^7^G modified RNA species^35–37^, and overexpression of m^7^G modified tRNAs ValAAC and LysCTT could rescue the downstream target expression and ESCC growth and progression, suggesting that METTL1 facilitates target mRNA translation and ESCC progression by regulating the m^7^G modification and expression of tRNAs but not other RNA species^37–40^. Our data uncovered the gene expression regulation mechanism that promotes the translation of oncogenic transcripts at the codon recognition step mediated by tRNA m^7^G modification.

The mTOR pathway is aberrantly activated in ESCC and promotes ESCC progression through negative regulation of autophagy^41,42^. Our data showed that depletion of METTL1 decreased the translation of the RPTOR mRNA and resulted in abnormal phosphorylation of ULK1, leading to the increased autophagy in ESCC cells. Overexpression of RPTOR, or knockdown of ULK1, successfully rescued the proliferation and progression of METTL1 knockdown ESCC cells, confirming that the RPTOR/ULK1 axis is an essential downstream target of METTL1 in the ESCC cells. The mTOR pathway is frequently overactivated and promotes mRNA translation and cancer progression in various types of cancers, and is a promising therapeutic target for cancer therapy^43,44^. Here we found that METTL1-mediated tRNA m^7^G modifications promote the translation of mTOR pathway components and therefore facilitate the mTOR activation and ESCC progression. Our data uncovered the mechanism underlying mTOR overactivation in cancers and suggested that targeting mTOR/ULK1/autophagy could be an effective strategy for METTL1 overactive ESCC treatment (Fig. 7n).

Overall, using ESCC clinical samples, cell culture, xenograft models, *Mettl1* conditional knockout and conditional knockin mice and in vivo ESCC initiation/progression models, we have demonstrated the strong physiological functions of METTL1/WDR4 mediated tRNA m^7^G modifications in selective oncogenic mRNA translation and ESCC progression. Our data uncovered the mechanism underlying METTL1-mediated ESCC tumorigenesis and provided molecular basis for the establishment of novel therapeutic strategies for ESCC treatment.

## Methods

### Patient samples

ESCC tumor and adjacent normal esophageal tissues from 120 esophageal carcinoma patients who underwent esophagectomy between September 2002 and July 2019 were obtained from the Sun Yat-Sen University Cancer Center (Guangzhou, China). Baseline information of patients was shown in Supplementary Table 2. Paraffin-embedded specimens were used for immunohistochemistry (IHC) analysis. Informed consents were obtained from all patients prior to analysis. All patient-related studies were reviewed and approved by the Institutional Review Board of the hospital (B2021-131-01). The datasets from The Cancer Genome Atlas (TCGA) and cBioPortal for Cancer Genomics were used for analysis of the expression and clinical association between METTL1/WDR4 and ESCC prognosis.

### Immunohistochemistry (IHC) staining

Immunohistochemistry staining was performed as previously described^45^. The following primary antibodies (anti-METTL1, Proteintech, 1:2000 dilution; Anti-WDR4, Abcam, 1:1000 dilution; Anti-Ki67, Proteintech, 1:8000 dilution; Anti-RPTOR, Proteintech, 1:500 dilution; Anti-pS6K1, Cell signaling technology, 1:1000 dilution; Anti-pULK1, Affinity, 1:1000 dilution; Anti-p4EBP1, Cell signaling technology, 1:1000 dilution) were used for detection of indicated protein expression. IHC staining was evaluated as H-score using QuPath (v0.2.3) by recording the staining intensity and the proportion of the cells with positive staining^46^. Tissues with H-score of ≧100 were defined as high expression samples, and tissues with H-score below 100 were defined as low expression samples. For survival analysis, patients were stratified into four groups based on their relative levels of H-scores: (>75%, 50–75%, 25–50%, and 0–25%).

### Immunofluorescence (IF) staining

The tissues were processed and incubated with primary antibodies (Anti-LC3a/b, Cell signaling technology, 1:200 dilution) following the IHC protocol, and the blocking buffer containing 5% normal donkey serum, 1% BSA, and 0.2% Triton X-100 was used in the blocking step. Next, the slides were incubated with secondary antibody (goat anti-rabbit IgG H&L DyLight® 594, Abcam, 1:100 dilution) at room temperature for 50 min, followed by treatment with 4, 6-diamidino-2-phenylindole at room temperature for 5 min. The slides were then examined and imaged under a microscope (ZEISS, Germany). The positive stained cells were counted with ImageJ (version 1.53n).

### Cell culture

KYSE150 and KYSE30 cells were purchased from Shanghai EK-Bioscience Biotechnology Co., Ltd. 293T cells were purchased from American Type Culture Collection (ATCC). All cells were cultured in DMEM (Gibco, USA) containing 10% fetal bovine serum (FBS, Gibco, USA), 1% Penicillin (Gibco, USA), and 1% streptomycin (Gibco, USA) in a water-saturated atmosphere under 5% CO_2_ at 37 °C in an incubator (Thermo Scientific, USA).

### Knockdown of METTL1 or WDR4 in ESCC cells

Lentiviral vectors expressing pLKO.1 shRNA targeting GFP (shGFP), METTL1 (shM1), and WDR4 (shW4) were purchased from Horizon Discovery. Inc. For lentivirus production, the lentiviral vectors, packaging vector pCMV-ΔR8.9, and enveloped vector pCMV-VSVG were co-transfected into 293T cells with Lipofectamine 2000 reagent (Invitrogen, USA). Forty-eight hours after the transfection, the packaged viruses were collected and used for infection of ESCC cells using 10 μg/ml Polybrene (Solarbio, China). 2.5 μg/ml puromycin (Solarbio, China) was used to select the infected cells for 48 h. METTL1 was knocked down in puromycin intake assay using small interfering RNAs (siRNAs) targeting the 3′-UTR of METTL1.

### Exogenous expression of proteins and tRNAs in ESCC cells

The full-length ORF of human METTL1 gene (NM_005371.6) and WDR4 gene (NM_005228.5) were cloned into pFLAG-CMV2 plasmid (Sigma, USA) to generate the METTL1 and WDR4 expression plasmids. The ORF of RPTOR gene (NM_001163034.2) was cloned into pEZ-Lv201 vector (GeneCopoeia, China). For stable overexpression, METTL1 ORF (NM_005371.6) was inserted into pCDH plasmid. Q5 Site-Directed Mutagenesis Kit (New England Biolabs) was used to develop the METTL1 catalytic dead mutant (aa160–163, LFPD to AFPA). The ValAAC or LysCTT tRNA expression plasmids were developed by cloning two repeats of ValAAC or LysCTT gene sequence and related regulatory elements into pUC19 vector. Lipofectamine 2000 reagent was used to transfect the plasmids into cells following the manufacturer’s instructions.

### RNA extraction and qRT-PCR

Trizol reagent (Invitrogen, USA) was used to isolate total RNAs following the manufacturer’s instructions. 2 μg RNA was used to perform reverse transcription by HiScript III RT SuperMix for qPCR Kit (Vazyme, China). Next, the cDNAs were 1:20 diluted followed by qRT-PCR using TB Green^TM^ Premix Ex Taq^TM^ II (Takara, Japan) in StepOnePlus^TM^ real-time PCR system (Thermo Scientific, USA). *β-ACTIN* was used as an internal control.

### tRNA mass spectrometry analysis

tRNAs were first purified from total RNAs by Urea-PAGE electrophoresis and size selection. Then the purified tRNAs were digested into single nucleosides, which were then analyzed by liquid chromatography-coupled mass spectrometry (LC-MS) using the Agilent 6460 QQQ mass spectrometer with an Agilent 1260 HPLC system. The levels of m^7^G tRNA modifications in different samples were calculated as ratio of m^7^G tRNA modification normalized peak area to the sum of the normalized peak area of all detected nucleosides in tRNAs.

### Western blot, northwestern blot, and northern blot

Western blot, northwestern blot, and northern blot assays were performed as previously reported^18,47,48^. Briefly, for northern blotting, 10% TBE-UREA gel was used to separate 2 μg RNAs by electrophoresis. Then the separated RNAs were transferred onto a positively charged nylon membrane followed by cross-linking with Ultraviolet (UV) light. The tRNAs and U6 snRNA were blotted with corresponding digoxigenin-labeled probes. For northwestern blotting, after transfer and cross-linking, the RNA-containing nylon membranes were blotted with anti-m^7^G antibody (MBL International, USA, 1:2000 dilution). Then the anti-m^7^G antibody signals were detected as described previously^47^. For anti-m^7^G methylation immunoprecipitation and tRNA northern blots, the total RNAs were incubated with anti-m^7^G antibody, then the immunoprecipitated RNAs were subjected to northern blot assay with indicated probes. Antibodies were listed in Supplementary Table 3.

### tRNA charging assay

tRNA charging assay was performed as previously described 18. Briefly, total RNAs were treated with Tris pH 9.5 (a final concentration of 0.1 M) at 37 °C for 60 min, then the treated RNAs were purified and dissolved in AcE storage buffer (10 mM sodium acetate (pH 5.0), 1 mM EDTA). Acid-Urea polyacrylamide gel Electrophoresis was performed to separate the Tris pH 9.5 treated RNAs, which then transferred to nylon membranes and blotted with indicated probes following the northern blot procedures.

### Colony-formation, cell proliferation assays

For the cell proliferation assay, 1500 ESCC cells were seeded into 96-well plates. Cell proliferation was analyzed for the following 5 days after planting using Cell Counting Kit-8 (Dojindo, Japan) following the manufacturer’s instructions. EdU cell proliferation assay was performed using the BeyoClick EdU Cell Proliferation Kit (Beyotime, China). For colony-formation assay, 1000 cells were seeded into 6-well plates and cultured for 10 days. The number of colonies stained with 0.5% crystal violet was then calculated with ImageJ (version 1.53n).

### Cell apoptosis assays

Cell apoptosis assay was performed using Annexin V-FITC Apoptosis Detection Kit (KeyGEN BioTECH, China) following the manufacturer’s instruction. The percentage of positive cells was detected using CytoFLEX (Beckman Coulter, USA). Flowjo (version 10) was used for analysis.

### Polysome profiling

Polysome profiling was performed as described previously^18^. Briefly, ESCC cells were incubated with 100 μg/ml cycloheximide for 3 min and then lysed using the polysome lysis buffer for 10 min on ice. After centrifuged at 13,000 × *g* for 10 min at 4 °C, 1 ml of supernatant was layered onto 11 ml 10–50% sucrose gradient and separated by centrifuging at 185,000 × *g* for 3 h at 4 °C. The samples were then fractionated and analyzed using the BR-188 Density Gradient Fractionation System (Brandel, USA).

### Puromycin intake assay

Cells were incubated with medium containing 1 μM puromycin for 30 min at 37 °C, and then subjected to protein isolation and western blotting. Anti-puromycin antibody (MABE343, Millipore, 1:50,000 dilution) was used to detect the new protein synthesis rate.

### tRNA reduction and cleavage sequencing (TRAC-seq)

TRAC-seq was performed as previously descripted^29^. Small RNAs were isolated from total RNA of ESCC cells using mirVana miRNA Isolation Kit (Invitrogen, USA). Next, recombinant wild-type AlkB and D135S AlkB proteins were used to remove the dominant methylations to facilitate the efficient reverse translation of tRNAs. Half of the demethylated RNAs were then treated with 0.1 M NaBH_4_ for 30 min on ice at dark, followed by treatment with aniline-acetate solution (H_2_O: glacial acetate acid: aniline, 7:3:1) at dark for 2 h to induce the m^7^G-site-specific cleavage. Then the RNA samples purified, together with the other half of the demethylated RNA samples were subjected to cDNA library construction with Multiplex Small RNA Library Prep Set for Illumina Kit (New England Biolabs, USA) for the high-throughput sequencing. The data were analyzed as previously described^29^.

### Polyribosome-bound mRNAs sequencing (polyribosome-seq)

Polyribosome-seq was performed as previously described^49^. Briefly, cells were incubated with 100 μg/ml cycloheximide for 3 min and then lysed with cell lysis buffer [1% Triton X-100 in ribosome buffer (RB buffer) [200 mM KCl, 15 mM MgCl_2_, 20 mM HEPES-KOH (pH 7.4), 100 μg/ml cycloheximide and 2 mM dithiothreitol] for 30 min on ice. After centrifuged at 16,200 × *g* for 10 min at 4 °C, the supernatants were transferred on the surface of 11 ml sucrose buffer (30% sucrose in RB buffer). Polyribosome-bound mRNAs were pelleted by ultra-centrifugation at 185,000 × *g* for 5 h at 4 °C and purified using TRIzol reagent (Invitrogen, USA). The cDNA library construction and sequencing were performed using BGISEQ-500 platform (BGI-Shenzhen, China). The gene expression level was normalized by using FPKM method. TEs were calculated by dividing FPKM in polyribosome-seq by FPKM in input RNA-seq.

### Ribosome profiling sequencing (ribo-seq)

Briefly, ESCC cells were incubated with DMEM containing 2 µg/ml harringtonine for 2 min, followed by treatment with 100 µg/ml cycloheximide for translation blocking. The cell extracts were treated with RNase I and DNase I and then selected with the size exclusion columns. The RNA Clean and Concentrator-25 kit (ZYMO Research, USA) was used for ribosome footprint fragments isolation and magnet beads (Vazyme, USA) were used for purification. Next, Ribo-seq libraries were generated using NEBNext® Multiple Small RNA Library Prep Set for Illumina® (New England Biolabs, USA). RiboToolkit (http://rnainformatics.org.cn/RiboToolkit/) was used for ribo-seq dada analysis^18,50^. TE was calculated by dividing the CDS footprinting FPKM by its mRNA FPKM.

### Gene ontology and pathway analysis

The differentially translated mRNAs identified in the polyribosome-seq data and ribosome profiling were used for gene ontology and pathway analysis using the ToppFun module of the ToppGene Suite (https://toppgene.cchmc.org/enrichment.jsp). The gene sets related to negative regulation of autophagy and mTOR signaling pathway were downloaded from GSEA portal (https://www.gsea-msigdb.org/gsea/index.jsp), and the translation matrix was analyzed using GSEA version 4.1 portal.

### Autophagic flux detection

The mRFP-GFP-LC3 adenoviral was obtained from HanBio Technology (Shanghai, China). ESCC cells were plated in 24-well plates and grew until 70% confluence, followed by incubation in DMEM with the adenoviruses for 2 h at 37 °C. LC3 puncta with yellow, green, or red fluorescent signal were detected with Zeiss LSM710 confocal microscope (Carl Zeiss) fitted with a ×60 oil immersion objective. After autophagosome fused with lysosome, the fluorescent signal of EGFP in autolysosomes could be quenched under the acidic condition inside the lysosome, while the RFP fluorescent signal was almost unaffected. Thus, autophagosomes and autolysosomes are shown as yellow puncta or red puncta respectively in green and red-merged images. Increased autophagic flux could be reflected by the increase of both yellow and red puncta in cells, and the blocked autophagic flux could be reflected in the case that only yellow puncta are increased without red puncta alteration, or that both yellow and red puncta are decreased in ESCC cells^51^.

### Animals

BALB/c Nude mice were obtained from Guangdong Medical Laboratory Animal Center (Foshan, China). Wild-type C57BL/6 mice were obtained from Model Animal Research Center of Nanjing University (Nanjing, China). The animal protocols were reviewed and approved by the Animal Care and Use Committee of Sun Yat-sen University (SYSU-IACUC-2021-000089, SYSU-IACUC-2021-000093). The study was in compliance with ethical regulations regarding Reporting of In Vivo Experiments (ARRIVE) guidelines. The Committee limits tumor growth to no more than 10% of the animal’s original body weight and the average tumor diameter to no more than 20 mm. Mice were maintained under controlled conditions (12/12 h light/dark cycle [lights on at 08.00 a.m.]; 23 ± 2 °C; 55 ± 10% humidity with food and water ad libitum). Mice were 6-week-old at the start of the experiments.

### Subcutaneous implantation mouse model

For subcutaneous implantation model, 5 × 10^6^ K30 cells were injected into randomly grouped 5-week-old female BALB/c Nude mice. The length (*a*) and width (*b*) of the tumors were measured at indicated time points using a caliper and the tumor volumes (*V*) were calculated by the formula *V* = 1/2 × *a* × *b*^2^. At the endpoint, mice were sacrificed and tumor tissues were collected and the weight of the tumors was measured.

### Establishment of epithelium-specific *Mettl1* conditional knockout mice

CRISPR-Cas9 technology was used to generate the *Mettl1* flox (fl) mice by the Beijing Biocytogen Co., Ltd. *Keratin14-CreER* mice were crossed with *Mettl1*^*fl/fl*^ mice to obtain epithelium-specific conditional *Mettl1* knockout mice (*Keratin14-CreER; Mettl1*^*fl/fl*^) for subsequent experiments. Mouse Tail Direct PCR Kit (Foregene, China) was used for genotyping. Primers were listed in Supplementary Table 4. Cre was activated by continuous three-day intraperitoneal (i.p.) injection of tamoxifen (Sigma, USA) at a dose of 9 mg per 40 g body weight per day.

### Establishment of epithelium-specific *Mettl1* conditional knockin mice

CRISPR-Cas9 technology was used to generate the *Mettl1* conditional knockin mice by the Beijing Biocytogen Co., Ltd. A CAG promoter driven loxP-Stop-loxP-Mettl1-IRES-tdTomato fragment was inserted into the Rosa26 locus. To generate the *Mettl1* conditional knockin mice (*Keratin14-CreER; Mettl1*^*ki*^), *Keratin14-CreER* mice were crossed with *Mettl1*^*ki*^ mice. Primers for transgenic genotyping were listed in Supplementary Table 4. Cre was activated as described above.

### In vivo ESCC tumorigenesis model

The in vivo ESCC tumorigenesis was induced using the DNA alkylating agent diethylnitrosamine (DEN)/sorafenib treatment method as previously described^32^. Briefly, 6-week-old mixed male and female *Mettl1* cKO, cKI, and their corresponding control C57BL/6 mice were treated with DEN (Sigma, USA) in sweetened drinking water (40 mg per 1000 ml) for 8 weeks. Sorafenib (MedChemExpress, USA) was administered by i.p. injection as a solution at 10 mg/ml dissolved in cottonseed oil containing 5% dimethylsulfoxide for 12 weeks (cKO group) or 10 weeks (cKI group). Injections of sorafenib were given on alternate days at a dose of 50 mg per 1000 g body weight.

### Hematoxylin and eosin stain (H&E) staining

Briefly, Paraffin-embedded esophageal tissues were cut into 5-μm-thick sections. Then the sections were baked for 1 h at 65 °C and dewaxed. The slides were washed with double-distilled water and stained with hematoxylin and eosin. Then, sections were dehydrated. Tumor grades of the ESCC mouse model were evaluated according to the H&E staining as follows: showing signs of normal appearance (normal); epithelial dysplasia confined to esophageal epidermis, unclearness of basement membrane (dysplasia); loss of the basement membrane, extensive invasion into muscle layer (ESCC).

### Quantification and statistical analysis

Quantitative data are shown as mean ± SD. Error bars in the scatterplots and the bar graphs represent SD. Exact *P* values in all cases are shown on graphs or provided in source data file. Two-tailed unpaired student’s *t* test, two-tailed Mann–Whitney U test, Kruskal–Wallis test, and one-way ANOVA with Dunnett’s or Tukey’s multiple comparison test or was used for statistical analysis unless stated elsewhere. Survival curves were compared with the log-rank test. Statistical analysis was performed using GraphPad Prism version 8.3.0 or SPSS version 25.

### Reporting summary

Further information on research design is available in the Nature Research Reporting Summary linked to this article.

## Supplementary information

Supplementary Information

Reporting Summary

## Data Availability

The accession number of raw sequencing data deposited to NCBI Gene Expression Omnibus is GSE169590. The TCGA data referenced during the study are available in a public repository from the TCGA website (https://www.cancer.gov/about-nci/organization/ccg/research/structural-genomics/tcga). Source data are provided with this paper.

## Source data

Source Data

## Code availability

All the open source codes were described in previous reports^29,50^. The custom codes were previously deposited in GitHub at https://github.com/rnabioinfor/TRAC-Seq, http://rnainformatics.org.cn/RiboToolkit/.
