# Supplementary material for: N7-methylguanosine tRNA modification promotes esophageal squamous cell carcinoma tumorigenesis via the RPTOR/ULK1/autophagy axis
Source: Nat Commun. 2022 Mar 18;13:1478. doi: 10.1038/s41467-022-29125-7 (PMC8933395; doi:10.1038/s41467-022-29125-7)
Supplement: Supplementary file 1 — Supplementary Information [file 41467_2022_29125_MOESM1_ESM.pdf]

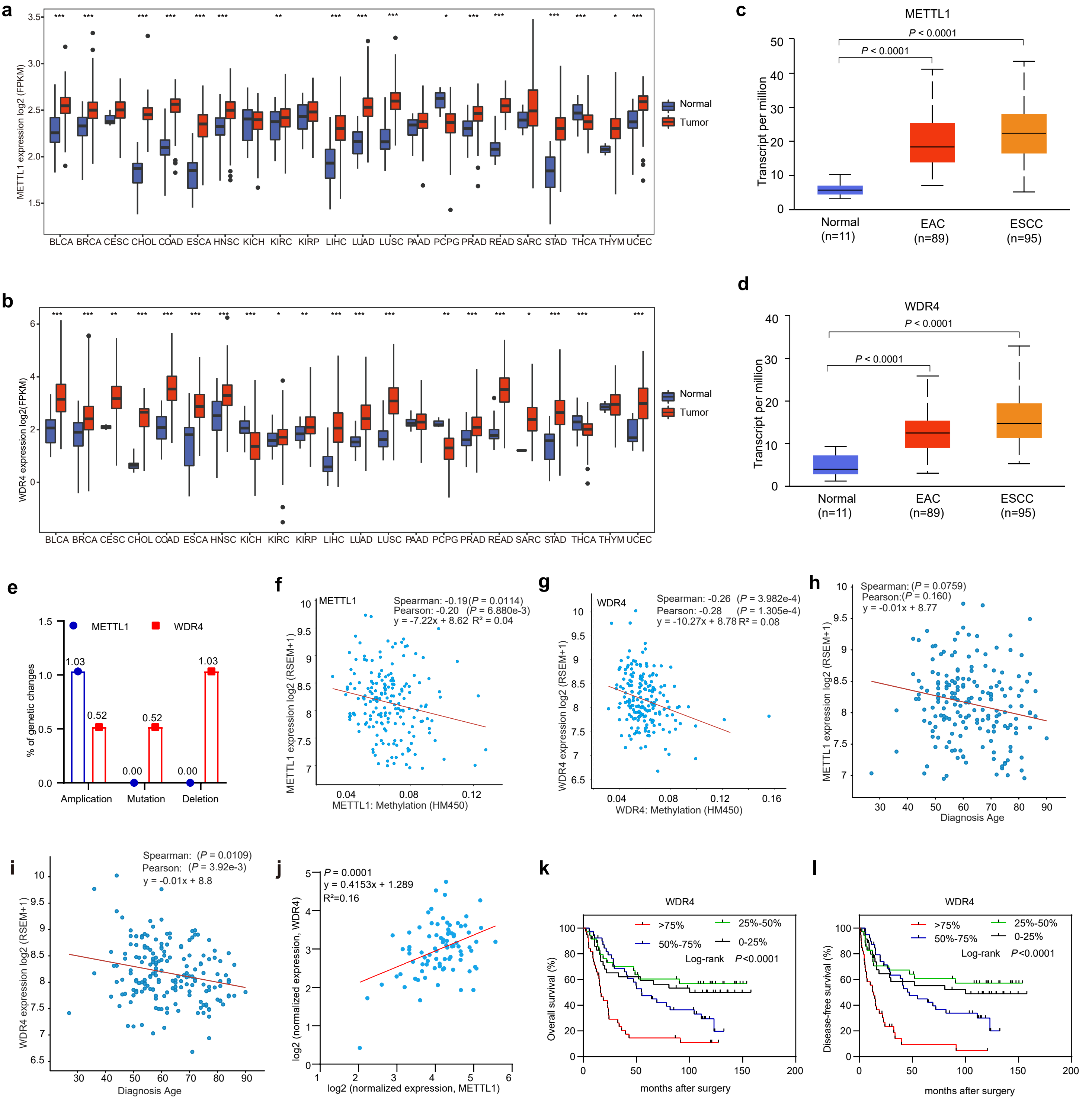

Supplementary Figure 1 Clinical association of METTL1 and WDR4 in ESCCs.
a-b, The relative mRNA levels of METTL1 (**a**) and WDR4 (**b**) in 22 cancer types in TCGA. **P* < 0.05, ***P* < 0.01, ****P* < 0.001.The exact *P* values and sample size for normal and tumor tissues were provided in a source data file. **c-d**, Quantification of METTL1 (**c**) and WDR4 (**d**) expression in normal esophageal tissues and esophageal tumors. **e**, Gene amplification, mutation, and depletion of METTL1 and WDR4 in ESCCs. **f-g**, Correlation between the mRNA expression and DNA methylation of METTL1 (**f**) and WDR4 (**g**) in ESCC. **h-i**, The correlation between patient age and METTL1 (**h**) or WDR4 (**i**) expression level. **j**, Correlation between the expression of METTL1 and WDR4 in ESCCs. **k-l**, Kaplan-Meier analysis of the overall survival (**k**) or disease-free survival (**l**) of ESCC patients based on different H-scores of WDR4. The box plots showing minima, maxima, centre, bounds of box and whiskers and percentile for (**a-d**). Data presented as mean ± SD. *P* values were calculated by two-tailed Mann-Whitney U test for (**a-d**), Pearson correlation analysis for (**f-i**) and log-rank test for (**k-l**). The data underlying **a-j** referenced during the study are available in a public repository from the TCGA website (https://www.cancer.gov/about-nci/organization/ccg/research/structural-genomics/tcga). The source data of k and l are protected and are not available due to data privacy laws.

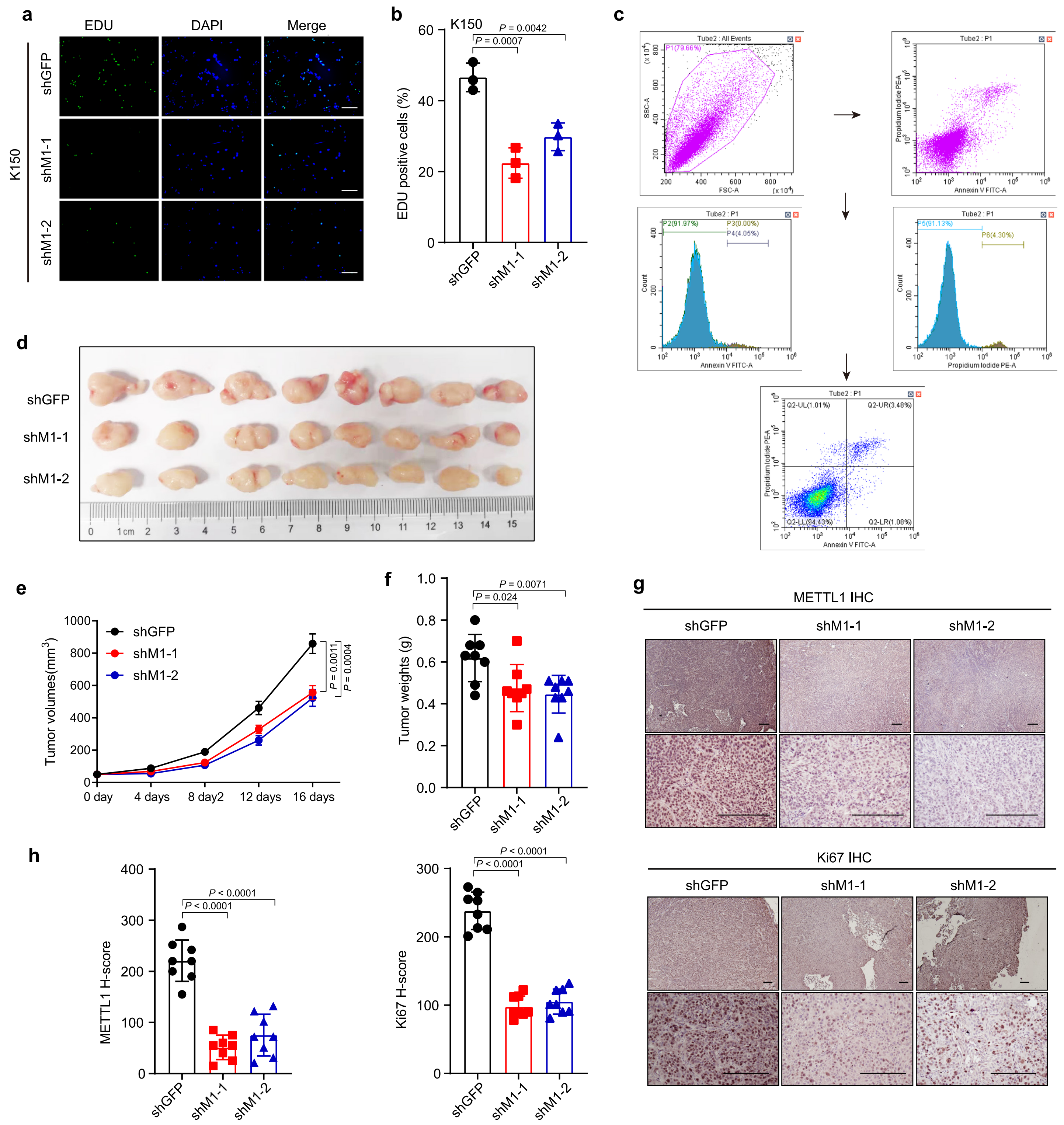

Supplementary Figure 2 METTL1 knockdown impairs ESCC progression in vitro and in xenograft model.
a-b, Edu assay (**a**) and the quantitative analysis (**b**) of METTL1 knockdown and control K150 ESCC cells. Scale bar, 200 μm. **c**, Example of the gating strategy used in the apoptosis assay. **d**, Overview of tumors in xenograft mice model subcutaneously implanted with METTL1 knockdown and control K30 ESCC cells. **e**, Growth curves of tumor volumes in METTL1 knockdown and control K30 ESCC cells. **f**, Tumor weights in METTL1 knockdown and control K30 ESCC cells. **g-h**, Representative images of METTL1 and Ki67 IHC staining (**g**) and the quantitative H-scores (**h**) of tumors obtained from the K150 xenograft model. Scale bar, 200 μm. Data represented as mean ± SD by one-way ANOVA with Dunnett's multiple comparison test from three independent experiments for (**b**) and eight biological independent samples for (**d-g**). Source data are provided as a Source Data file.

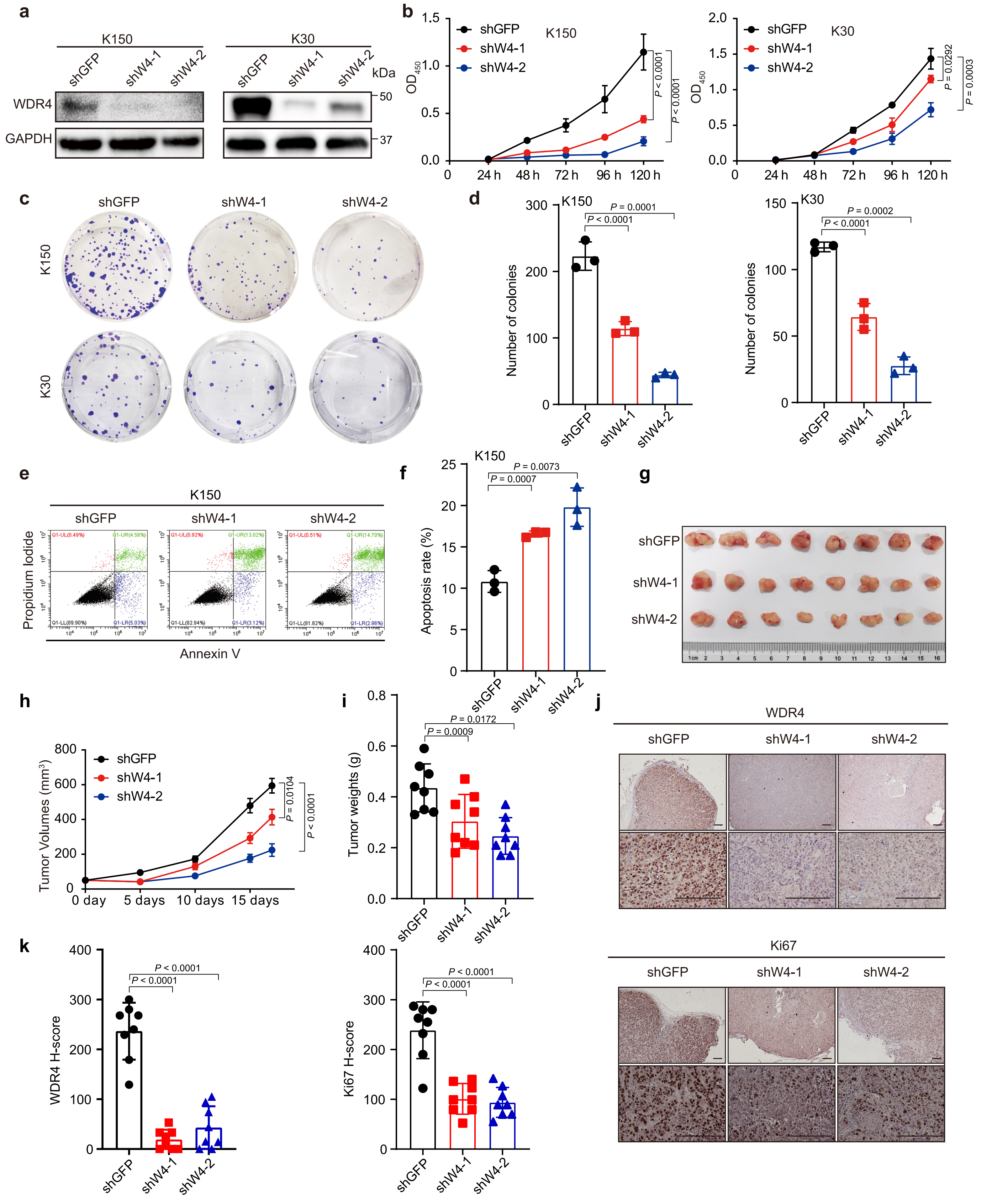

Supplementary Figure 3 WDR4 knockdown impairs ESCC progression in vitro and in vivo.
a, Western blot with indicated antibodies in WDR4 knockdown and control ESCC cells. **b**, CCK8 analysis of WDR4 knockdown and control ESCC cells. **c-d**, Colony-formation assay (**c**) and the quantification analysis (**d**) of WDR4 knockdown and control ESCC cells. **e-f**, Apoptosis assay (**e**) and the quantification analysis (**f**) of WDR4 knockdown and control ESCC cells. **g**, Overview of tumors in xenograft model subcutaneously implanted with of WDR4 knockdown and control K150 ESCC cells. **h**, Growth curves in xenograft model injected with WDR4 knockdown and control K150 ESCC cells. **i**, Tumor weights in xenograft model injected with WDR4 knockdown and control K150 ESCC cells. **j-k**, Representative images of WDR4 and Ki67 IHC staining (**j**) and the quantitative H-scores (**k**) of tumors obtained from the xenograft mice model. Scale bar, 200 μm. Data represented as mean ± SD by one-way ANOVA with Dunnett's multiple comparison test from three independent experiments for (**b**, **d**, and **f**) and eight biological samples for (**h-k**). Source data are provided as a Source Data file.

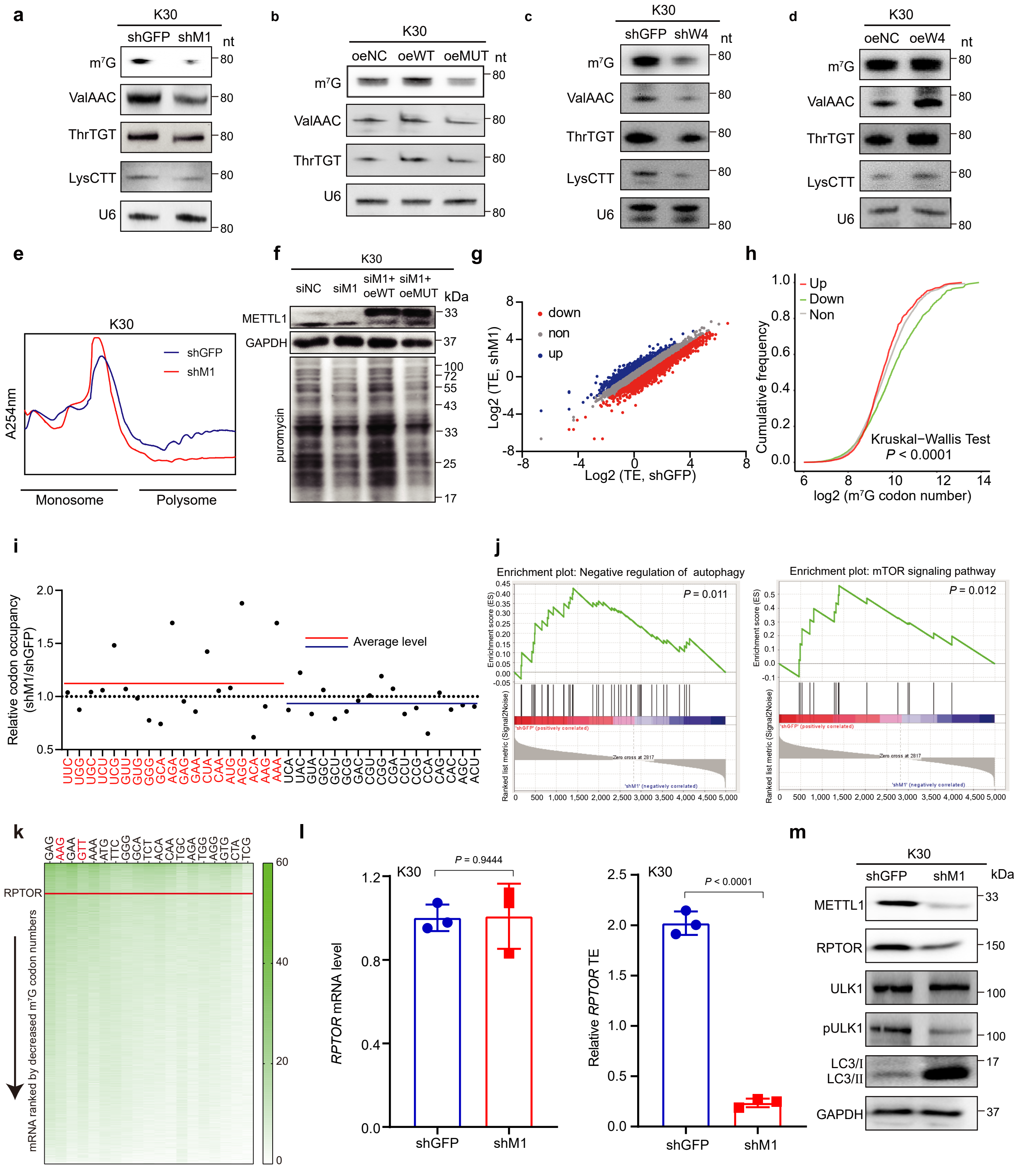

Supplementary Figure 4 METTL1 regulates m^7G tRNA modification, tRNA expression and oncogenic mRNA translation.
a-d, Northern blot with indicated tRNA probes in METTL1 depleted K30 cells (**a**), wild type METTL1 or mutant METTL1 overexpressed K30 cells (**b**), WDR4-depleted K30 cells (**c**) and WDR4 overexpressed K30 cells (**d**). U6 snRNA was used as a loading control. **e**, Polysome profiling of METTL1 depleted and control K30 cells. **f**, Puromycin intake assay of METTL1 depleted K30 cells rescued with wild type or catalytic inactive METTL1. **g**, Scatterplot of TEs in the METTL1 knockdown and control cells in the Ribo-seq data. **h**, Codon number in the coding sequence (CDS) region of the genes with increased TEs (up), decreased TEs (down), and other genes (non) in Ribo-seq data in the METTL1 knockdown cells. **i**, Relative ribosome occupancy at individual codons at A sites in METTL1 depleted cells and control cells. Only codons with detectable corresponding tRNA expression were shown. The codons decoded by non-m^7G tRNAs (black) and codons decoded by m^7G tRNAs (red) were presented. **j**, GSEA analysis of decreased translated mRNAs in Ribo-seq data. **k**, Heatmap showed the enrichment of m^7G-related codons in all detected mRNAs. **l**, qRT-PCR analysis of polyribosome-mRNAs and input mRNAs in K30 cells from three independent experiments. **m**, Western blot analysis with indicated antibodies in K30 cells. Data represented as mean ± SD. *P* values are calculated by permutation test for (**j**) and two-tailed unpaired Student's t test for (**i**). Source data are provided as a Source Data file.

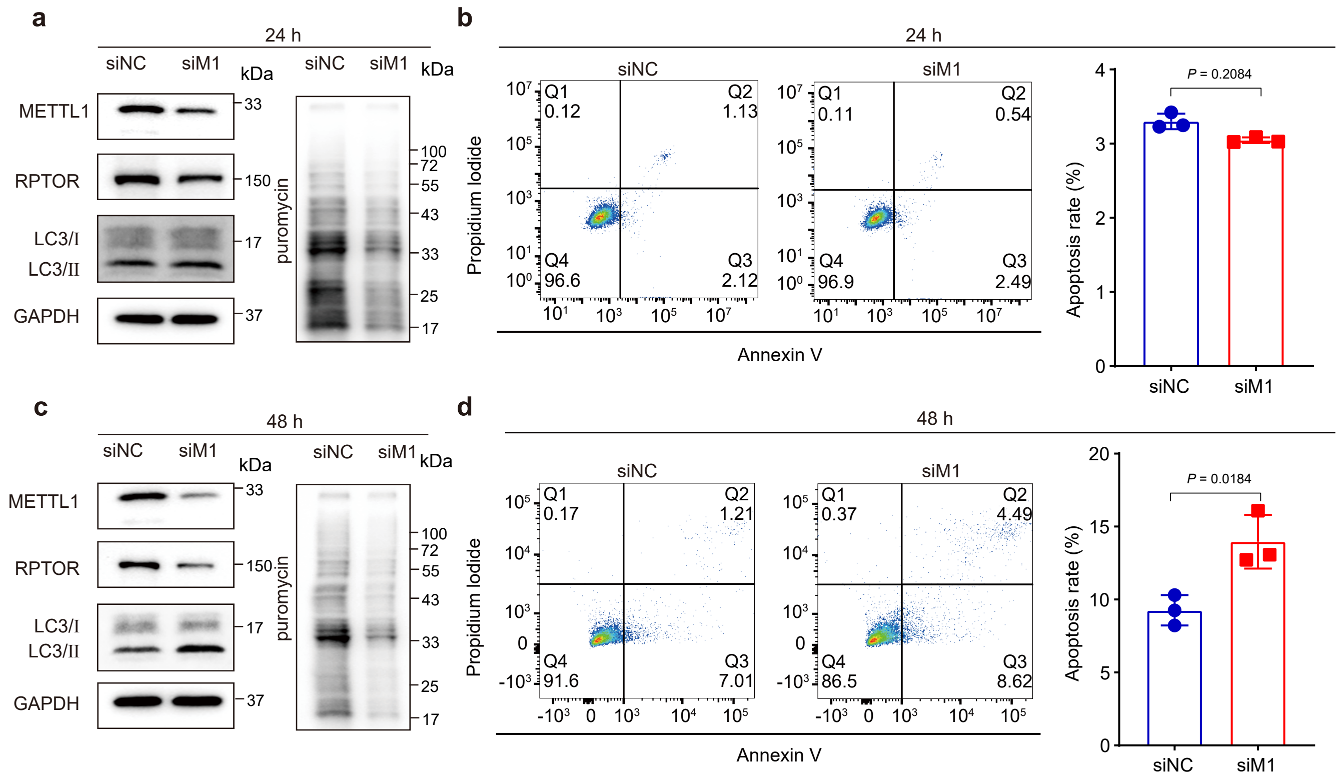

Supplementary Figure 5 Decreased mRNA translation upon METTL1 knockdown leads to impaired cell survival.
a, The puromycin incorporation and protein levels of RPTOR and LC3 at 24 h after METTL1 knockdown in K150 cells. **b**, The apoptosis rate of K150 ESCC cells at 24 h after METTL1 knockdown. **c**, The puromycin incorporation and protein levels of RPTOR and LC3 at 48 h after METTL1 knockdown in K150 cells. **d**, The apoptosis rate of K150 ESCC cells at 48 h after METTL1 knockdown. Data represented as mean ± SD by two-tailed unpaired Student's t test from three independent experiments for (**b** and **d**). Source data are provided as a Source Data file.

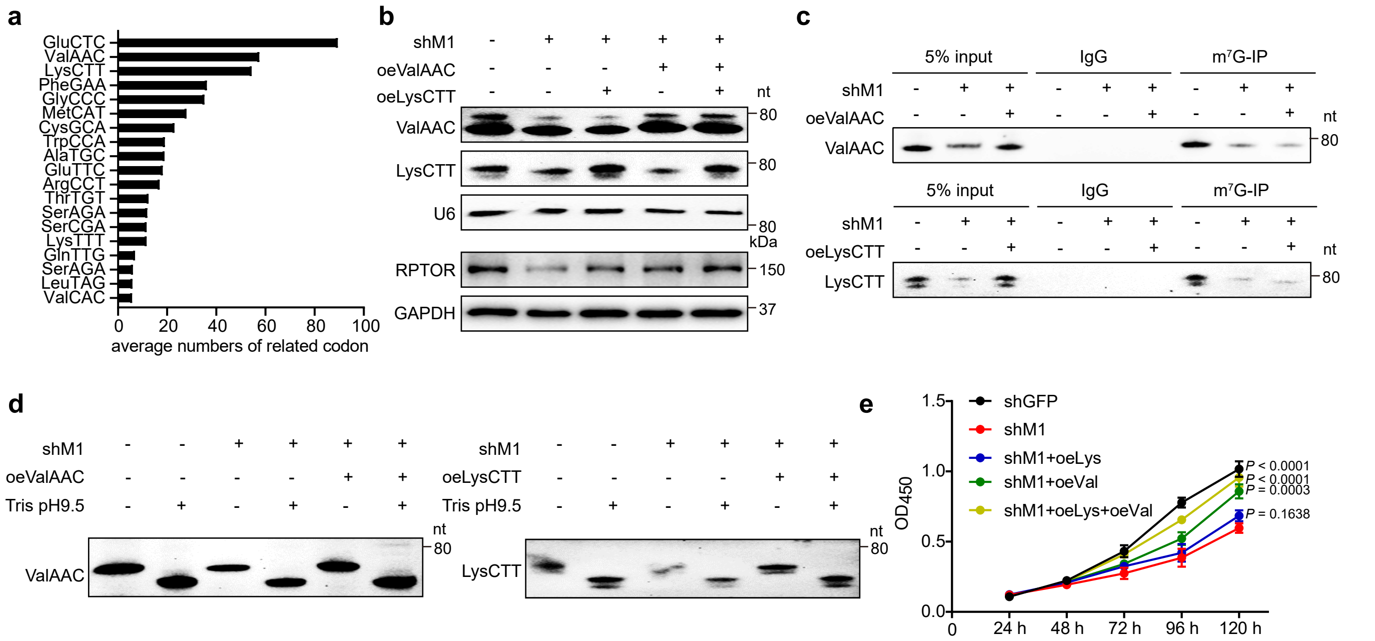

Supplementary Figure 6 m^{7}G-modified tRNAs mediate METTL1's function in ESCC progression.
a, Average number of individual m^{7}G-modified tRNA decoded codons in TE-down mRNAs in K150 ESCC cells. **b**, Northern blot and western blot with indicated probes and antibodies in K150 ESCC cells. **c**, m^{7}G MeRIP-northern blot with indicated probes. **d**, tRNA charging assay to detect the charging of ectopically overexpressed ValAAC and LysCTT tRNAs. **e**, CCK8 assay of METTL1 knockdown K150 ESCC cells with or without tRNA overexpression. Data represented as mean ± SD from three independent experiments for (**e**). Significant difference from shM1 group calculated by one-way ANOVA with Dunnett's multiple comparison test are shown on graphs (**e**). Source data are provided as a Source Data file.

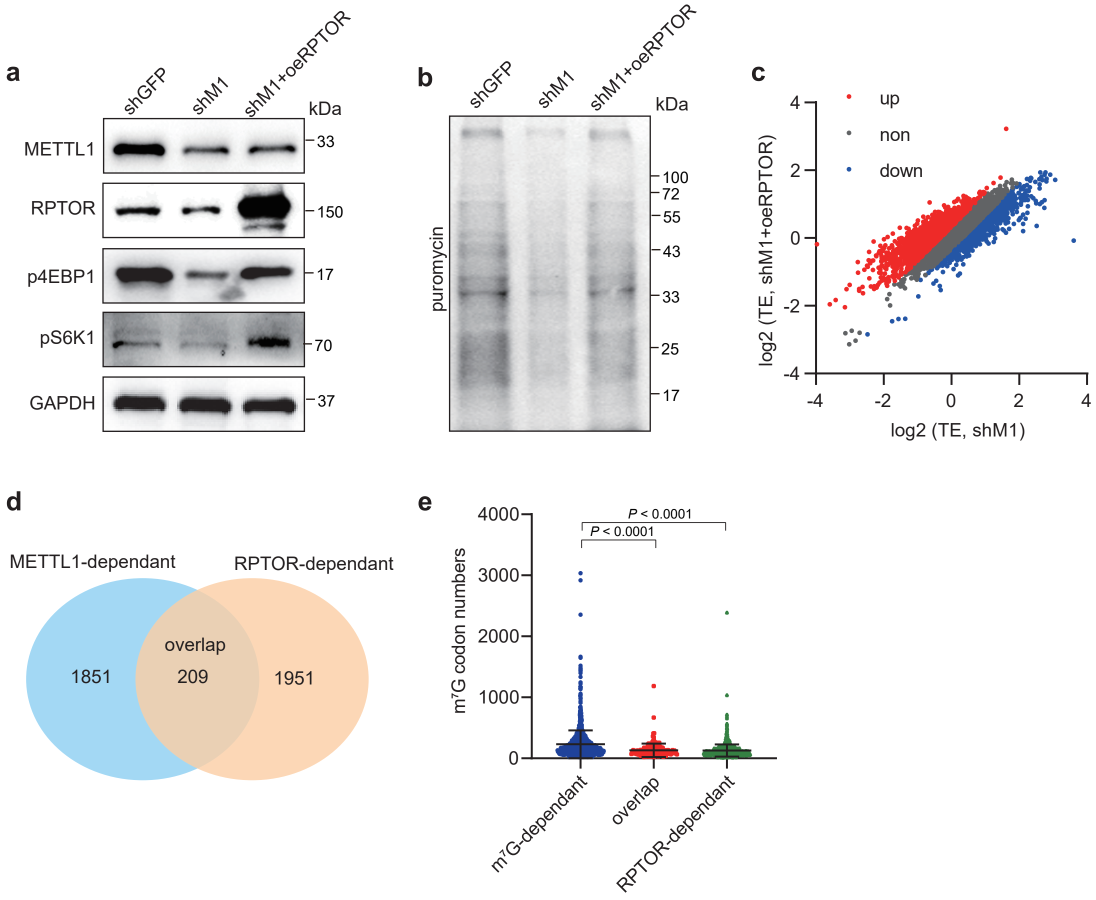

Supplementary Figure 7 Overexpression of RPTOR restores translation of METTL1 depleted ESCC cells.
a, Western blot with indicated antibodies in METTL1 depleted K150 ESCC cells with or without RPTOR overexpression. **b**, Puromycin incorporation assay in METTL1 depleted K150 ESCC cells with or without RPTOR overexpression. **c**, Scatterplot of TEs in the METTL1 knockdown cells with or without RPTOR overexpression. **d**, Overlap of TE down mRNAs upon METTL1 knockdown and TE up mRNAs upon RPTOR overexpression. **e**, Comparison of m^7G related codon numbers in METTL1-dependent, RPTOR-dependent and the overlap group. Data represented as mean ± SD by two-tailed Mann-Whitney U test from three biological independent samples. Source data are provided as a Source Data file.

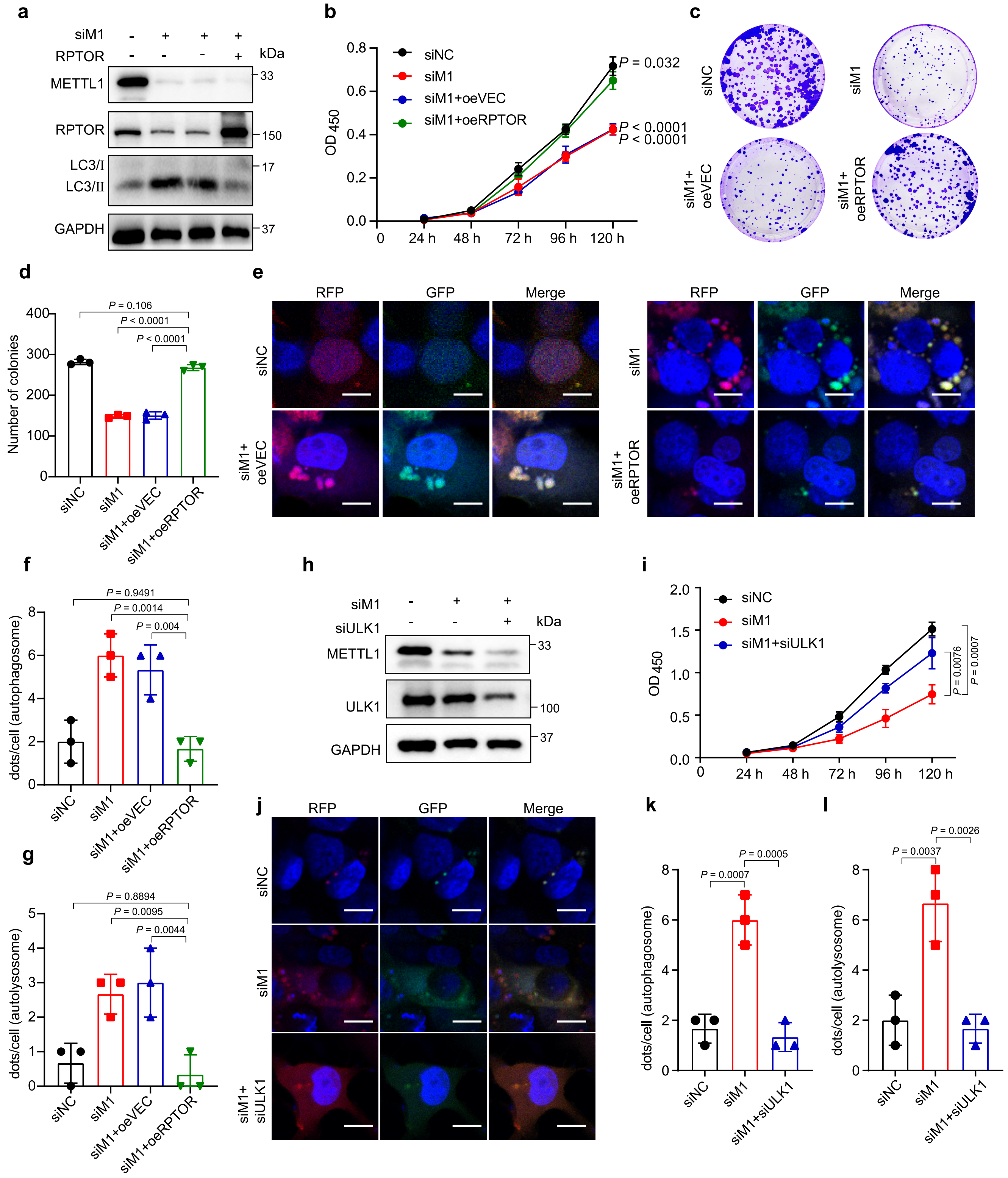

Supplementary Figure 8 RPTOR and ULK1-mediated autophagy play essential roles in ESCC progression in K30 cells.
a, Western blot analysis with indicated antibodies in METTL1 knockdown K30 cells with or without RPTOR overexpression. **b**, CCK8 assay of METTL1 knockdown K30 cells with or without RPTOR overexpression. **c-d**, Colony-formation assay (**c**) and the quantification analysis (**d**) of METTL1 knockdown K30 cells with or without RPTOR overexpression. **e-g**, The autophagic fluxes (**e**) and the quantification analysis (**f**, **g**) of K30 cells stably expressed mRFP-EGFP-LC3 fusion protein. Scale bar, 10 μm. **h**, Western blot analysis with indicated antibodies in K30 cells. **i**, CCK8 assay of METTL1 knockdown K30 cells with or without ULK1 knockdown. **j-l**, The autophagic fluxes (**j**) and the quantification analysis (**k**, **l**) of K30 cells stably expressed mRFP-EGFP-LC3 fusion protein. Scale bar, 10 μm. Data represented as mean ± SD from three independent experiments. Significant difference from siM1+oeRPTOR group calculated by one-way ANOVA with Dunnett's multiple comparison test are shown on graphs (**b**, **d**, **f**, and **g**), and significant difference from siM1+siULK1 group calculated by one-way ANOVA with Dunnett's multiple comparison test are shown on graphs (**i**, **k**, and **l**). Source data are provided as a Source Data file.

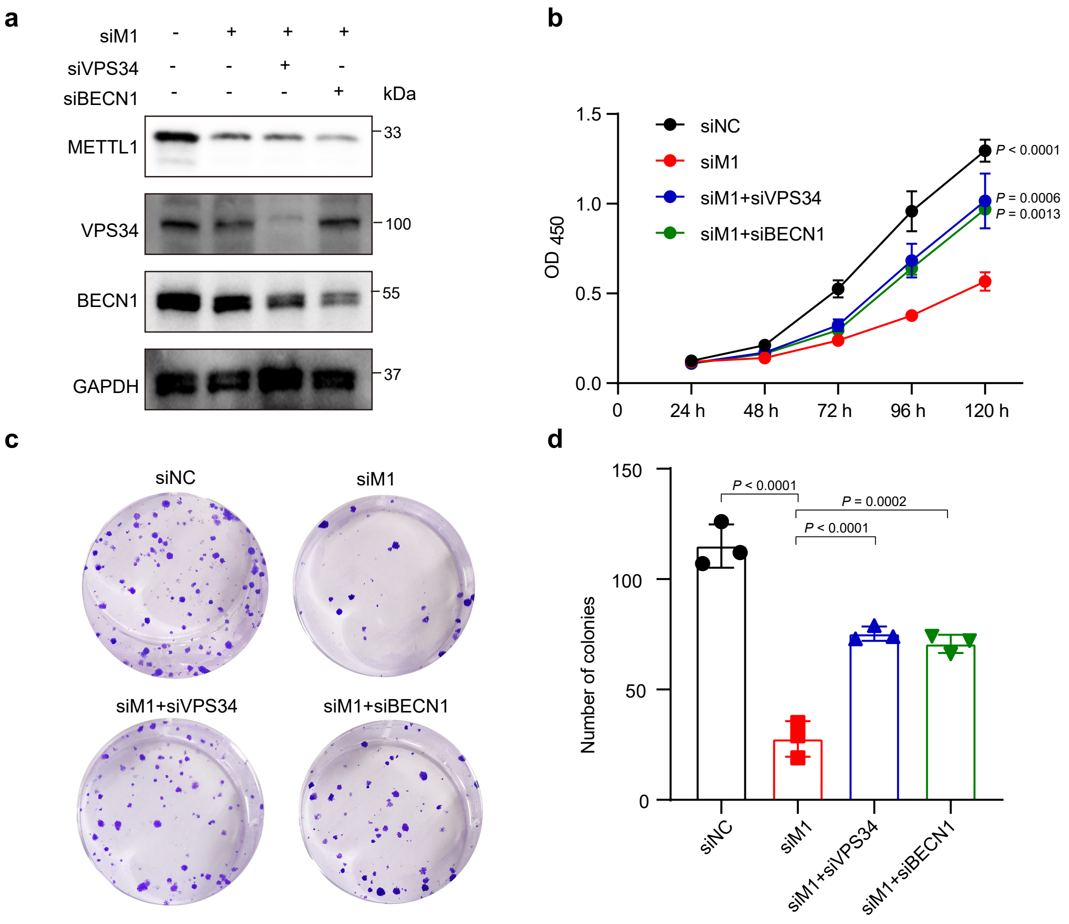

Supplementary Figure 9 VPS34 and BECN1 mediate METTL1's function in ESCC.
a, Western blot analysis with indicated antibodies. **b**, CCK8 assay of METTL1 knockdown K150 cells with VPS34 or BECN1 knockdown. **c**-**d**, Colony-formation assay (**c**) and the quantitative analysis (**d**) of METTL1 knockdown K150 cells with VPS34 or BECN1 knockdown. Data represented as mean ± SD from three independent experiments. Significant difference from siM1 group calculated by one-way ANOVA with Dunnett's multiple comparison test are shown on graphs. Source data are provided as a Source Data file.

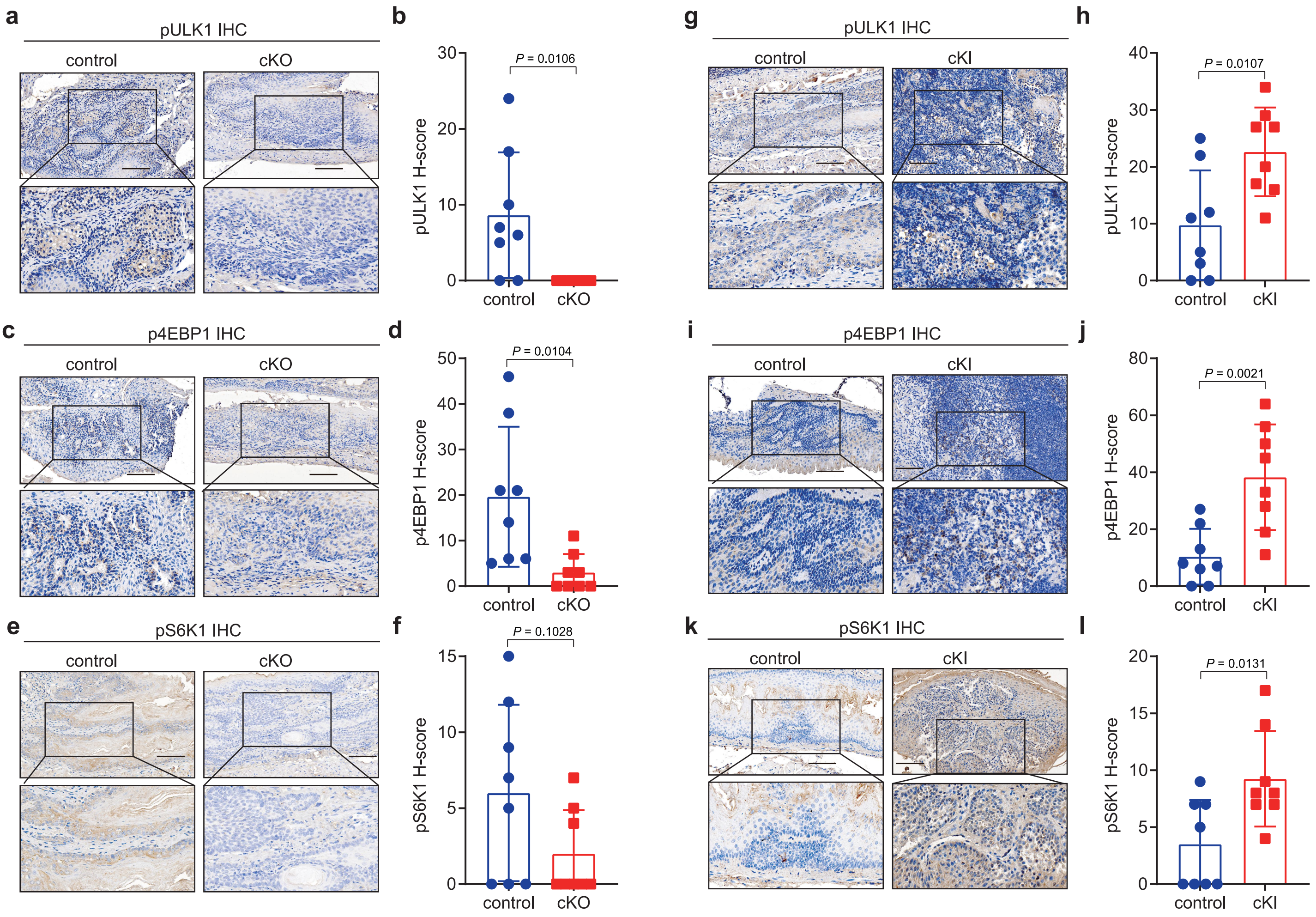

Supplementary Figure 10 The downstream targets of RPTOR were regulated by METTL1 in vivo.
a-b, Representative images (**a**) and the quantification (**b**) of pULK1 IHC staining in *Mettl1* cKO and control mice. Scale bar, 100 μm. **c-d**, Representative images (**c**) and the quantification (**d**) of p4EBP1 IHC staining in *Mettl1* cKO and control mice. Scale bar, 100 μm. **e-f**, Representative images (**e**) and the quantification (**f**) of pS6K1 IHC staining in *Mettl1* cKO and control mice. Scale bar, 100 μm. **g-h**, Representative images (**g**) and the quantification (**h**) of pULK1 IHC staining in *Mettl1* cKI and control mice. Scale bar, 100 μm. **i-j**, Representative images (**i**) and the quantification (**j**) of p4EBP1 IHC staining in *Mettl1* cKI and control mice. Scale bar, 100 μm. **k-l**, Representative images (**k**) and the quantification (**l**) of pS6K1 IHC staining in *Mettl1* cKI and control mice. Scale bar, 100 μm. Data represented as mean ± SD by two-tailed unpaired Student's t test.from eight biological independent samples for each group. Source data are provided as a Source Data file.

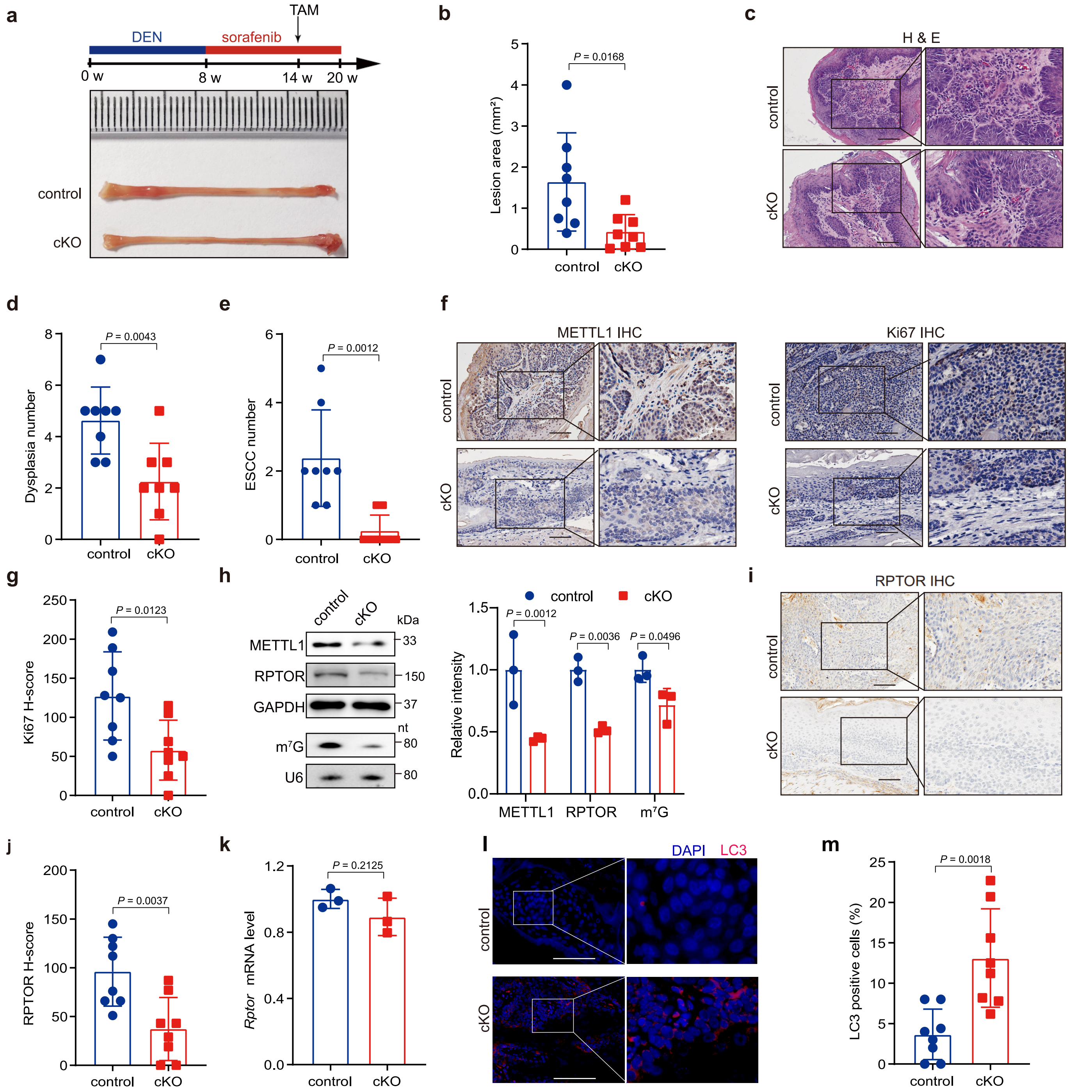

Supplementary Figure 11 Knockout of *Mettl1* inhibits in vivo ESCC progression.
a, Experimental design for ESCC progression model and the representative images of esophageal lesions in *Mettl1* cKO and control mice. **b**, Quantification of lesion areas. **c**, Representative H&E staining of esophagus. Scale bar, 100 μm. **d**, Quantification of dysplasia numbers. e, Quantification of ESCC numbers. **f-g**, Representative images of METTL1 and Ki67 IHC staining (**f**) and the quantification Ki67 H-scores (**g**) in *Mettl1* cKO and control mice. Scale bar, 100 μm. **h**, western blot analysis and northwestern blot analysis showed the m^{7}G modification level and indicated protein levels, right panel: quantification of the blot signals. **i-j**, Representative images (**i**) and the quantification (**j**) of RPTOR IHC staining. Scale bar, 100 μm. **k**, qRT-PCR analysis of *Rptor* mRNA level in the cKO and control mice. **l-m**, IF assay (**l**) and the quantification (**m**) of LC3 level. Scale bar, 100 μm. Data represented as mean ± SD by two-tailed unpaired Student's t test from eight biological independent samples for each group for (**a-g**, **i**, **j**, **l**, and **m**) and three biological independent samples for (**h** and **k**). Source data are provided as a Source Data file.

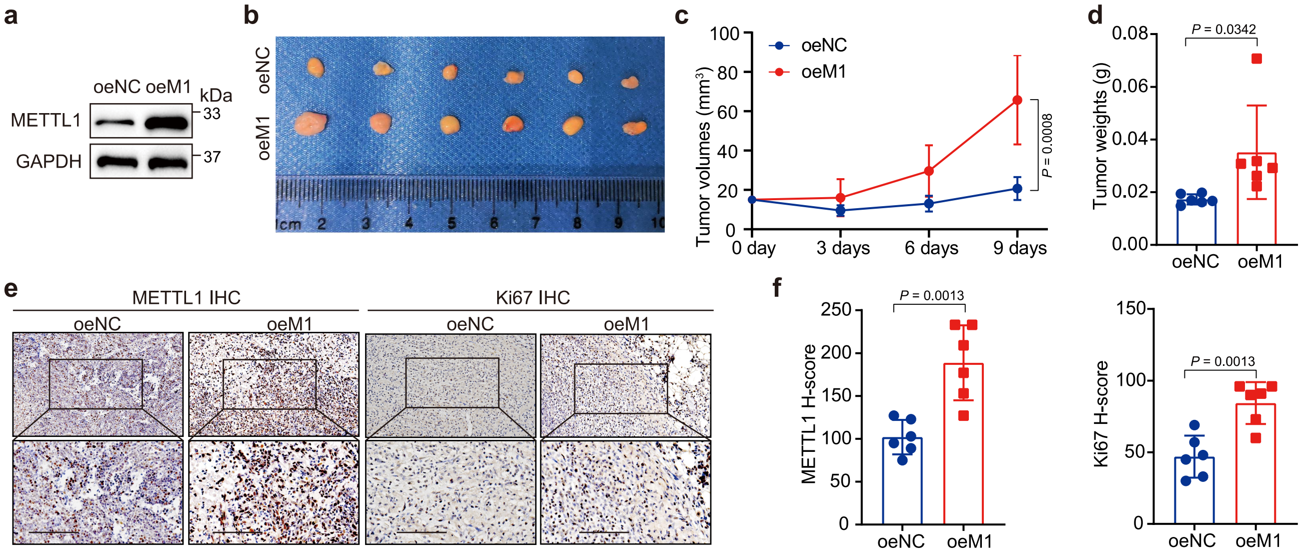

Supplementary Figure 12 METTL1 overexpression promotes ESCC tumorigenesis in xenograft mouse model.
a, Western blot assay showed the indicated protein levels in K150 ESCC cells. **b**, Overview of tumors in xenograft mice model subcutaneously implanted with METTL1 overexpression and control ESCC cells. **c**, Growth curves of tumor volumes in METTL1 overexpression and control group. **d**, Tumor weights in METTL1 overexpression and control group. **e-f**, Representative images of METTL1 and Ki67 IHC staining (**e**) and the quantitative H-scores (**f**) of tumors obtained from the xenograft model. Scale bar, 200 μm. Data represented as mean ± SD by two-tailed unpaired Student's t test from six biological independent samples for each group. Source data are provided as a Source Data file.

Supplementary Table 1. Genes enriched in autophagy and mTOR signaling pathway

**Genes enriched in autophagy pathway**
SRC
SREBF1
WDR81
DAPK3
ATG2A
ITGB4
QSOX1
BOK
RPTOR
WDR24
TMEM150A
CHMP6
KAT5
CAPN1
UBXN6
KLHL22
EEF1A2
ULK3
ATP6V0D1
MAP1S
CTSD
GPSM1
**Genes enriched in mTOR signaling pathway**
SREBF1
RPTOR
SFN
PML
POLDIP3
PRR5
EEF2

Supplementary Table 2. Baseline information of 120 patients with esophageal cancer.

Characteristic	Number
**Gender**	
Female	33 (27.5%)
Male	87 (72.5%)
**Age**	
Median (range)	58 (38-80)
**T category**	
T1	2 (1.7%)
T2	30 (25%)
T3	88 (73.3%)
T4	0 (0%)
**N category**	
N0	67 (55.8%)
N1	51 (42.5%)
N2	2 (1.7%)
**Overall stage**	
I	3 (2.5%)
II	73 (60.8%)
III	44 (36.7%)
IV	0 (0%)

Supplementary Table 3. Antibodies used in this study.

Antibodies	source	identifier
Rabbit monoclonal anti-WDR4	Abcam	Cat# ab169526
Rabbit polyclonal anti-Ki67	Abcam	Cat# ab15580
Rabbit polyclonal anti-Phospho-ULK1 (ser758)	Affinity	Cat# AF4387
Rabbit polyclonal anti-ULK1	Beyotime	Cat# AF8307
Phospho-4E-BP1 (Thr37/46) (236B4) Rabbit mAb	Cell signaling technology	Cat# 2855
Phospho-p70 S6 Kinase (Thr421/Ser424) Antibody	Cell signaling technology	Cat# 9204
Anti-rabbit IgG HRP-linked Antibody	Cell signaling technology	Cat# 7074S
Anti-mouse IgG HRP-linked Antibody	Cell signaling technology	Cat# 7076S
Rabbit polyclonal anti-LC3	Cell signaling technology	Cat# 2775S
Mouse monoclonal 7-methylguanosine (m^7G) antibody	MBL International	Cat# RN017M
Mouse monoclonal anti-puromycin	Millipore	Cat# MABE343
PI3 Kinase p110 Alpha Monoclonal antibody	Proteintech	Cat# 67071-1-Ig
Rabbit polyclonal anti-METTL1	Proteintech	Cat# 14994-1-AP
Mouse monoclonal anti-RPTOR	Proteintech	Cat# 20984-1-AP
Rabbit polyclonal anti-GAPDH	Proteintech	Cat# 10494-1-AP
Rabbit polyclonal anti-VPS34	Proteintech	Cat# 12452-1-AP
Rabbit polyclonal anti-BECN1	Proteintech	Cat# 11306-1-AP
Goat anti-rabbit IgG H&L DyLight® 594	Abcam	Cat# ab96885
Rabbit monoclonal anti-AKT	Proteintech	Cat#60203-2-Ig
Rabbit monoclonal anti-mTOR	Proteintech	Cat#66888-1-Ig

Supplementary Table 4. Oligonucleotides sequences in this study.

**The sequences of METTL1 shRNA**	
**No.**	**sequence (5' - 3')**
shMETTL1-1	AAATGAGTGCACATCCAGTCG
shMETTL1-2	TTTCTTATCCTTTGGGTCATC
shWDR4-1	AAGAGGCTGTCATCATCACTG
ShWDR4-2	TCTAACAGCATAGACAGGTGC
**The sequences of siRNA**	
**No.**	**sequence (5' - 3')**
METTL1-1	GATGACCCAAAGGATAAGAAA
METTL1-2	GGATGTGCACTCATTTCGA
ULK1-1	CCTTCGCGGTGGTCTTCAAdTdT
ULK1-2	CGCGGTACCTCCAGAGCAAdTdT
**Probe sequences of tRNAs and U6 snRNA**	
**No.**	**sequence (5' - 3')**
tRNA-Lys-CTT	AACGTGGGGCTCGAACCCAC
tRNA-Val-AAC	TGTTTCCGCCCGGTTTCGAA
tRNA-Thr-TGT	CTAACCCCTGAGCTATGGAG
U6 snRNA	TGGAACGCTTCACGAATTTG
**Primers used for the qRT-PCR assay**	
**No.**	**sequence (5' - 3')**
hGAPDH-F	GTCTCCTCTGACTTCAACAGCG
hGAPDH-R	ACCACCCTGTTGCTGTAGCCAA
hβ-ACTIN-F	CACCATTGGCAATGAGCGGTTC
hβ-ACTIN-R	AGGTCTTTGCGGATGTCCACGT
hmTOR-F	AGCATCGGATGCTTAGGAGTGG
hmTOR-R	CAGCCAGTCATCTTTGGAGACC
hRPTOR-F	GATCGTCAACAGCTATCACACGG
hRPTOR-R	CGAGTCGAAGTTCTGCCAGATC
hMETTL1-F	GGCTTCCAGAACATCGCCTGT
hMETTL1-R	TGTCCGCTTGAAATGTGGGTCG
hWDR4-F	CGTCTACTCCTTTTCGGTGCTG
hWDR4-R	GCAGTGAGGATGAAGCGGTCAT
hAKT1-F	TGGACTACCTGCACTCGGAGAA
hAKT1-R	GTGCCGCAAAAGGTCTTCATGG
hPIK3C2A-F	CTTACTCATTGCTTCACCAGTGG
hPIK3C2A-R	GCCTCAATCCAGGTCACAGCTA
hPIK3CA-F	GAAGCACCTGAATAGGCAAGTCG
hPIK3CA-R	GAGCATCCATGAAATCTGGTCGC

hPIK3CB-F	GGTAATCGGAGGATAGGGCAGT
hPIK3CB-R	CGGCAGTATGCTTCAAGGATGAC
mGapdh-F	CATCACTGCCACCCAGAAGACTG
mGapdh-R	ATGCCAGTGAGCTTCCCGTTCAG
mRaptor-F	CTTCCTATCCGTCTTGGCAGAC
mRaptor-R	CTCCAGACAGATGGCAATCAGG
mMettl1-F	GTGGAGTTTGCAGACATAGGCTG
mMettl1-R	GCACATAGTCCGACACCTTCAC
Firefy-Luc-F	GGTACTGTTGGTAAAGCCAC
Firefy-Luc-R	CTCTTCATAGCCTTATGCAG
Renilla-Luc-F	CAATGGGCAGGTGTCCACTC
Renilla-Luc-R	GTTCTGGATCATAAACTTTC
**Primers for floxed Mettl1 knockdown/knockin allele genotyping**	
**No.**	**Sequence (5’ - 3’)**
Keratin14-CreER-1	ACTGGGATCTTCGAACTCTTTGGA C
Keratin14-CreER-2	GATGTTGGG GCACTGCTCATTCACC
Keratin14-CreER-3	CCATCTGCCACCAGCCAG
Keratin14-CreER-4	TCGCCATCTTCCAGCAGG
METTL1-cKO-F	ATACACACTCCT GGCCACTCC TTGT
METTL1-cKO-R	CCCTGGCTT CTA GGGTGTGAA TCAG
METTL1-cKI-WT-F	AGTCGCTCTGAGTTGTTATCAG
METTL1-cKI-WT-R	TGA GCATGTCTTTAATCTACCTCGATG
METTL1-cKI-MUT-F	AGTCGCTCTGAGTTGTTATCAG
METTL1-cKI-MUT-R	GTCAATGGAAAGTCCCTATTGGCGT
