# Supplementary material for: N7-methylguanosine tRNA modification promotes esophageal squamous cell carcinoma tumorigenesis via the RPTOR/ULK1/autophagy axis
Source: Nat Commun. 2022 Mar 18;13:1478. doi: 10.1038/s41467-022-29125-7 (PMC8933395; doi:10.1038/s41467-022-29125-7)
Supplement: Supplementary file 2 — Reporting Summary [file 41467_2022_29125_MOESM2_ESM.pdf]

nature research

Corresponding author(s): Shuibin Lin

Last updated by author(s): 17/2/22

Reporting Summary

Nature Research wishes to improve the reproducibility of the work that we publish. This form provides structure for consistency and transparency in reporting. For further information on Nature Research policies, see our Editorial Policies and the Editorial Policy Checklist.

Statistics

For all statistical analyses, confirm that the following items are present in the figure legend, table legend, main text, or Methods section.

n/a	Confirmed	
☐	☒	The exact sample size (n) for each experimental group/condition, given as a discrete number and unit of measurement
☐	☒	A statement on whether measurements were taken from distinct samples or whether the same sample was measured repeatedly
☐	☒	The statistical test(s) used AND whether they are one- or two-sided<br>*Only common tests should be described solely by name; describe more complex techniques in the Methods section.*
☐	☒	A description of all covariates tested
☐	☒	A description of any assumptions or corrections, such as tests of normality and adjustment for multiple comparisons
☐	☒	A full description of the statistical parameters including central tendency (e.g. means) or other basic estimates (e.g. regression coefficient) AND variation (e.g. standard deviation) or associated estimates of uncertainty (e.g. confidence intervals)
☐	☒	For null hypothesis testing, the test statistic (e.g. F, t, r) with confidence intervals, effect sizes, degrees of freedom and P value noted<br>*Give P values as exact values whenever suitable.*
☒	☐	For Bayesian analysis, information on the choice of priors and Markov chain Monte Carlo settings
☒	☐	For hierarchical and complex designs, identification of the appropriate level for tests and full reporting of outcomes
☐	☒	Estimates of effect sizes (e.g. Cohen's d, Pearson's r), indicating how they were calculated

Our web collection on statistics for biologists contains articles on many of the points above.

Software and code

Policy information about availability of computer code

Data collection	Immunohistochemistry and Immunofluorescence images were captured using Zeiss Zen Blue edition (version 3.4). ImageJ (version 1.53n) was used to count the numbers of colonies. All the open source codes were described in previous reports (Lin, S., Liu, Q., Jiang, Y. Z. & Gregory, R. I. Nucleotide resolution profiling of m(7)G tRNA modification by TRAC-Seq. Nat Protoc. 2019; Liu, Q., Shvarts, T., Sliz, P. & Gregory, R. I. RiboToolkit: an integrated platform for analysis and annotation of ribosome profiling data to decode mRNA translation at codon resolution. Nucleic Acids Res).
Data analysis	H-score of the immunohistochemistry were analyzed using QuPath (version 0.2.3). The numbers of colonies in colony-formation assay, proportion of positive-stained cells in the immunofluorescence assay, intensity of blot bands were analysed with ImageJ (version 1.53n). Statistical analysis was performed using GraphPad Prism version 8.3.0 or SPSS version 25. Flowjo was used for the apoptosis assay analyses. The custom codes were previously deposited in GitHub at https://github.com/rnabioinfor/TRAC-Seq, http://rnainformatics.org.cn/RiboToolkit/.

For manuscripts utilizing custom algorithms or software that are central to the research but not yet described in published literature, software must be made available to editors and reviewers. We strongly encourage code deposition in a community repository (e.g. GitHub). See the Nature Research guidelines for submitting code & software for further information.

Data

Policy information about availability of data

All manuscripts must include a data availability statement. This statement should provide the following information, where applicable:

- Accession codes, unique identifiers, or web links for publicly available datasets
- A list of figures that have associated raw data
- A description of any restrictions on data availability

The data availability statement was provided in the manuscript.
Raw sequencing data are being submitted to NCBI Gene Expression Omnibus (GSE169590). The TCGA data referenced during the study are available in a public repository from the TCGA website (https://www.cancer.gov/about-nci/organization/ccg/research/structural-genomics/tcga).

Field-specific reporting

Please select the one below that is the best fit for your research. If you are not sure, read the appropriate sections before making your selection.

☒ Life sciences ☐ Behavioural & social sciences ☐ Ecological, evolutionary & environmental sciences

For a reference copy of the document with all sections, see nature.com/documents/nr-reporting-summary-flat.pdf

Life sciences study design

All studies must disclose on these points even when the disclosure is negative.

Sample size	For CCK8 assay, colony formation assay, EDU assay, Flow cytometry assay, Autophagic flux assay, three independent experiments were performed. For EDU assay, at least 200 cells were counted at three independent experiments. The xenograft mouse model experiments and the C57BL/6 experiments were performed with n = 8 biological independent samples for each group. Where possible, sample sizes were chosen based on established protocols in previous publications and/or generally-accepted criteria in the scientific community.
Data exclusions	No data exclusion was performed. However, data resulted from technical errors were excluded for data analysis. For example, for the polysome profiling assay, the abnormal absorbance values caused by the inevitable vibration of the instrument were excluded for data analysis, and dose not affect the conclusion. These exclusion criteria were pre-established.
Replication	Repetitive biologically independent experiments were done to confirm consistency of results. All attempts at replication were successful for those experiments. Sample sizes and number of biologically independent experiments with consistent results are indicated in the corresponding figure legends.
Randomization	The allocation was random.
Blinding	For immunohistochemistry, the quantitative analysis was performed using QuPath (version 0.2.3) with fixed parameters in a same experiment. For immunoImmunofluorescence, the quantitative analysis was performed using ImageJ (version 1.53n) with fixed parmeters in a same experiment. For apoptosis assay, the quantitative analysis was performed using Flowjo (version 10) with fixed gating strategies. For mersurement of numbers of colonies and intensity of blot bands, ImageJ (version 1.53n) was used with fixed gating strategies. With the application of these softwares, we have largely avoided the influence of human eyes and subjectivity on the conclusion in this study.

Reporting for specific materials, systems and methods

We require information from authors about some types of materials, experimental systems and methods used in many studies. Here, indicate whether each material, system or method listed is relevant to your study. If you are not sure if a list item applies to your research, read the appropriate section before selecting a response.

Materials & experimental systems

n/a	Involved in the study	
☐	☒	Antibodies
☐	☒	Eukaryotic cell lines
☒	☐	Palaeontology and archaeology
☐	☒	Animals and other organisms
☐	☒	Human research participants
☒	☐	Clinical data
☒	☐	Dual use research of concern

Methods

n/a	Involved in the study	
☒	☐	ChIP-seq
☐	☒	Flow cytometry
☒	☐	MRI-based neuroimaging

Antibodies

Antibodies used	The antibodies used in this study were listed in Supplementary Table 3.

Validation

Rabbit monoclonal anti-WDR4（Abcam, Cat# ab169526） has been previously used in our previous publications (Shuibin Lin et al., 2018, and Zihao Dai et al., 2021) and validated by the manufacturer (more information is available at https://www.abcam.cn/wdr4-antibody-epr11052-ab169526.html) and cited at least once.
Rabbit polyclonal anti-METTL1 (Proteintech, Cat# 14994-1-AP) has been previously used in our previous publications (Zihao Dai et al., 2021) and previously validated by the manufacturer (more information is available at https://www.ptglab.com/Products/METTL1-Antibody-14994-1-AP.htm) and cited at least 8 times.
Rabbit polyclonal anti-Ki67 (Abcam, Cat# ab15580) has been previously used in our previous publications (Zihao Dai et al., 2021) and validated by the manufacturer (more information is available at https://www.abcam.cn/ki67-antibody-ab15580.html) and cited at least 2858 times.
Mouse monoclonal 7-methylguanosine (m7G) antibody(MBL International, Cat# RN017M) has been previously used in our previous publications (Shuibin Lin et al., 2018, and Zihao Dai et al., 2021) and validated by the manufacturer (more information is available at https://www.mblbio.com/bio/g/dtl/A/index.html?pcd=RN017M) and cited at least 8 times.
Rabbit polyclonal anti-Phospho-ULK1 (ser758) (Affinity, Cat# AF4387) has been previously validated by the manufacturer (more information is available at http://affbiotech.cn/goods-14736-AF4387-Phospho_ULK1_Ser757_Ser758_Antibody.html) and cited at least 6 times.
Rabbit polyclonal anti-ULK1 (Beyotime, Cat# AF8307) was validated by knockdown ULK1 in K150 and K30 cells with siRNA (siULK1) and detecting experimentally induced changes in target antigen expression.
Phospho-4E-BP1 (Thr37/46) (236B4) Rabbit mAb(Cell signaling technology, Cat# 2855) has been previously validated by the manufacturer (more information is available at https://www.cellsignal.cn/products/primary-antibodies/phospho-4e-bp1-thr37-46-236b4-rabbit-mab/2855) and cited at least 1019 times.
Phospho-p70 S6 Kinase (Thr421/Ser424) Antibody (Cell signaling technology, Cat# 9204) has been previously validated by the manufacturer (more information is available at https://www.cellsignal.cn/products/primary-antibodies/phospho-p70-s6-kinase-thr421-ser424-antibody/9204?site-search-type=Products&N=4294956287&Ntt=9204&fromPage=plp&_requestid=12631) and cited at least 357 times.
Anti-rabbit IgG HRP-linked Antibody (Cell signaling technology, Cat# 7074S) has been previously validated by the manufacturer (more information is available at https://www.cellsignal.cn/products/secondary-antibodies/anti-rabbit-igg-hrp-linked-antibody/7074?site-search-type=Products&N=4294956287&Ntt=7074s&fromPage=plp&_requestid=12683) and cited at least 7233 times.
Anti-mouse IgG HRP-linked Antibody (Cell signaling technology, Cat# 7076S) has been previously validated by the manufacturer (more information is available at https://www.cellsignal.cn/products/secondary-antibodies/anti-mouse-igg-hrp-linked-antibody/7076?site-search-type=Products&N=4294956287&Ntt=7076s&fromPage=plp&_requestid=12742) and cited at least 4028 times.
Rabbit polyclonal anti-LC3 (Cell signaling technology, Cat# 2775S) has been previously validated by the manufacturer (more information is available at https://www.cellsignal.cn/products/primary-antibodies/lc3b-antibody/2775?site-search-type=Products&N=4294956287&Ntt=2775s&fromPage=plp&_requestid=12801) and cited at least 1402 times.
Mouse monoclonal anti-puromycin (Millipore, Cat# MABE343) has been previously used in our previous publications (Zihao Dai et al., 2021) and validated by the manufacturer (more information is available at https://www.sigmaaldrich.cn/CN/zh/search/mabe343?focus=products&page=1&perPage=30&sort=relevance&term=MABE343&type=product) and cited at least 378 times.
PI3 Kinase p110 Alpha Monoclonal antibody (Proteintech, Cat# 67071-1-Ig) has been previously validated by the manufacturer (more information is available at https://www.ptgcn.com/Products/PIK3CA-Antibody-67071-1-Ig.htm) and cited at least 34 times.
Mouse monoclonal anti-RPTOR (Proteintech, Cat# 20984-1-AP) has been previously validated by the manufacturer (more information is available at https://www.ptglab.com/Products/RPTOR-Antibody-20984-1-AP.htm) and cited at least 22 times.
Rabbit polyclonal anti-GAPDH (Proteintech, Cat# 10494-1-AP) has been previously validated by the manufacturer (more information is available at https://www.ptglab.com/products/GAPDH-Antibody-10494-1-AP.htm) and cited at least 3021 times.
Rabbit polyclonal anti-VPS34 (Proteintech, Cat# 12452-1-AP) has been previously validated by the manufacturer (more information is available at https://www.ptglab.com/products/PIK3C3-Antibody-12452-1-AP.htm) and cited at least 33 times.
Rabbit polyclonal anti-BECN1 (Proteintech, Cat# 11306-1-AP) has been previously validated by the manufacturer (more information is available at https://www.ptglab.com/products/BECN1-Antibody-11306-1-AP.htm) and cited at least 485 times.
Goat anti-rabbit IgG H&L DyLight® 594 (Abcam, Cat# ab96885) has been previously validated by the manufacturer (more information is available at https://www.abcam.cn/goat-rabbit-igg-hl-dylight-594-ab96885.html) and cited at least 22 times.
Rabbit monoclonal anti-AKT (Proteintech, Cat#60203-2-Ig) has been previously validated by the manufacturer (more information is available at https://www.ptglab.com/products/AKT-Antibody-60203-2-Ig.htm) and cited at least 91 times.
Rabbit monoclonal anti-mTOR (Proteintech, Cat#66888-1-Ig) has been previously validated by the manufacturer (more information is available at https://www.ptglab.com/Products/MTOR-Antibody-66888-1-Ig.htm) and cited at least 68 times.

Eukaryotic cell lines

Policy information about cell lines

Cell line source(s)

KYSE150 and KYSE30 cells were purchased from Shanghai EK-Bioscience Biotechnology Co., 293T cells were purchased from ATCC.

Authentication

All cell lines used were authenticated vis STR profiling.

Mycoplasma contamination

All cell lines tested negative for mycoplasma contamination.

Commonly misidentified lines
(See ICLAC register)

No cell line in this study was commonly misidentified.

Animals and other organisms

Policy information about studies involving animals; ARRIVE guidelines recommended for reporting animal research

Laboratory animals	Female BALB/c nude mice and mixed male and female C57BL/6 mice were used in this study. C57BL/6 mice were 6-week old at the start of the experiments and BALB/c Nude mice were 5-week-old at the start of experiments.
Wild animals	The study did not involve wild animals.
Field-collected samples	The study did not invole samples collected from field.
Ethics oversight	All animal care and experiment protocols were approved by the Animal Care and Use Committee of Sun Yat-sen University(SYSU-IACUC-2021-000089, SYSU-IACUC-2021-000093). The study was in compliance with all relevant ethical regulations regarding Reporting of In Vivo Experiments (ARRIVE) guidelines.

Note that full information on the approval of the study protocol must also be provided in the manuscript.

Human research participants

Policy information about studies involving human research participants

Population characteristics	ESCC tumor and adjacent esophageal tissues from 120 esophageal carcinoma patients (33 female and 87 male aged 37-80) who underwent esophagectomy between September 2002 and July 2019 were obtained from Sun Yat-Sen University Cancer Center (Guangzhou, China). Baseline information of patients was shown in Supplementary Table 2.
Recruitment	ESCC tumor and adjacent esophageal tissues from 120 esophageal carcinoma patients who underwent esophagectomy between September 2002 and July 2019 were obtained from Sun Yat-Sen University Cancer Center (Guangzhou, China). We used available samples for this study. there is no self-selection bias or other biases are present.
Ethics oversight	Acquisition of all clinical samples was approved by the the clinical research ethics committee of Sun Yat-sen University Cancer Center (B2021-131-01).

Note that full information on the approval of the study protocol must also be provided in the manuscript.

Flow Cytometry

Plots

Confirm that:

☒ The axis labels state the marker and fluorochrome used (e.g. CD4-FITC).

☒ The axis scales are clearly visible. Include numbers along axes only for bottom left plot of group (a 'group' is an analysis of identical markers).

☒ All plots are contour plots with outliers or pseudocolor plots.

☒ A numerical value for number of cells or percentage (with statistics) is provided.

Methodology

Sample preparation	The samples were preparation following the manufacturer’s instruction using Annexin V-FITC Apoptosis Detection Kit (KeyGEN BioTECH, China).
Instrument	CytoFLEX (Beckman Coulter, USA)
Software	Flowjo software was used for analysis.
Cell population abundance	Cell populations were determined by the the size and granularity of the cells respectively estimated by forward and side scatter, as well as the corresponding posivie or negative signal.
Gating strategy	The data expressed in a histogram and two peaks were shown which can be interpreted as the positive and negative dataset.

☒ Tick this box to confirm that a figure exemplifying the gating strategy is provided in the Supplementary Information.
